# Disease-associated genetic variants can cause missense effects in tissue-specific protein isoforms

**DOI:** 10.1038/s41467-026-74280-w

**Published:** 2026-06-16

**Authors:** Giovanna Weykopf, Mihaly Badonyi, Elias T. Friman, Jasmine Minh Hang Nguyen, Alexis Ioannou, Benjamin J. Livesey, Audrey Coutts, Elizabeth F. Hird, Murray Wham, Chloe M. Stanton, Veronique Vitart, Jing Su, Lee Murphy, J. Kenneth Baillie, Mark D. Gorrell, Joseph A. Marsh, Wendy A. Bickmore, Simon C. Biddie

**Affiliations:** 1 MRC Human Genetics Unit, Institute of Genetics and Cancer, University of Edinburgh, Edinburgh, UK; 2 Liver Enzymes in Metabolism and Inflammation, Centenary Institute, Sydney, NSW Australia; 3 The University of Sydney, Faculty of Medicine and Health, Sydney, NSW Australia; 4 Clinical Research Facility Genetics Core, University of Edinburgh, Edinburgh, UK; 5 NHS Lothian, Edinburgh, UK; 6 Bioinformatics analysis core, Institute of Genetics and Cancer, University of Edinburgh, Edinburgh, UK; 7 Institute for Regeneration and Repair, University of Edinburgh, Edinburgh, UK; 8 Baillie Gifford Pandemic Science Hub, Centre for Inflammation Research, University of Edinburgh, Edinburgh, UK; 9 Roslin Institute, University of Edinburgh, Edinburgh, UK; 10 Intensive Care Unit, Royal Infirmary of Edinburgh, Edinburgh, UK

**Keywords:** Gene expression profiling, Transcriptomics, Protein structure predictions, Rare variants

## Abstract

Genetic variants can cause protein-coding mutations that result in disease. Variants are typically interpreted using the reference transcript for a gene. However, most human multi-exon genes have alternative isoforms. We show that, consistent with their reduced evolutionary constraint, coding exons in alternative isoforms harbour more population variants than exons of reference isoforms, and that these variants are more likely to cause nonsynonymous mutations. Common and rare disease-associated variants mapping to alternative transcripts can lead to amino acid substitutions predicted to be structurally damaging in the corresponding protein isoform. The alternative transcripts to which disease-associated variants map demonstrate high tissue-specificity, with many unannotated in reference human genomes, and only revealed by long-read RNA-sequencing. As an example, we report an unannotated, alternative transcript of the inflammasome regulator DPP9 that is lung epithelium-specific, that harbours a common genetic variant associated with severe COVID-19 and lung fibrosis. Using deep RNA sequencing of full-length transcript isoforms by targeted capture, we confirm the expression of the unannotated *DPP9* isoform. The DPP9 isoform variant causes a p.Leu8Pro missense mutation in an alternative first exon, predicted to disrupt the encoded alpha helix, and we show that the variant alters DPP9 enzymatic activity. Our findings highlight the importance of considering alternative isoforms, their tissue-specific expression, and full-length transcripts in variant interpretation, with implications for uncovering underappreciated mechanisms of both common and rare disease.

## Abstract (web summary)

Isoforms are seldom considered in genetic variant interpretation. Here the authors map common and rare variants to alternative isoforms, which can be highly expressed and tissue-specific. A common DPP9 variant maps to a lung expressed alternative isoform, with the missense variant altering activity.

## Introduction

Transcript isoforms are alternative mRNAs produced from a single gene locus, through mechanisms such as alternative splicing or the use of alternative promoters^1^. Approximately 95% of human multi-exon genes generate multiple transcript isoforms, thereby expanding protein diversity^2^. Protein-coding alternative isoforms show high tissue-specificity^3^ and can be developmental stage-specific or context-dependent^4^. Alternative isoforms can have functions different than the main isoform, including gain-of-function or dominant-negative properties^5^. For example, transcription factor isoforms can have differential binding activities^6^, while immune receptor isoforms can have dominant-negative effects on the immune response^7^.

Rare genetic variants that alter isoform expression or sequence contribute to disease. For example, by disrupting isoform balance or intron inclusion, splice site mutations can lead to developmental and neuropsychiatric disorders^8,9^. However, most clinical interpretation of coding variants remains limited to the reference isoform^10^, and only isolated cases of pathogenic variants specific to alternative isoforms have been reported. One example is a missense mutation in an adult-specific isoform of *SCN5A*, associated with cardiovascular conductive disease^11^. Recent advances in long-read RNA sequencing have revealed previously unannotated transcripts and highlighted the extent of tissue-specific isoform expression^3,12^. Thus, there is a need to re-examine disease-associated mutations in this broad isoform landscape.

Although many alternative transcripts are detected, the majority may not encode stable or functional proteins. For most highly expressed protein-coding genes, one isoform predominates expression^13^, and many alternative isoforms lack cross-species conservation or signs of purifying selection^14,15^. Analyses of human genetic variation similarly suggest that most alternative exons evolve under relaxed constraint^16^. These observations imply that, while some alternative isoforms may be biochemically competent, they are often not required for function and may represent non-essential or non-adaptive transcriptional byproducts. A key unmet need is to determine which genetic variants exert their effects through specific alternative isoforms, particularly those expressed in restricted tissues or developmental stages.

Here, we address this challenge by integrating large protein language model-based variant effect scores, AlphaFold structural modelling and tissue-specific long-read transcriptomic data to prioritize alternative isoforms expressed in relevant biological contexts. We analyzed common and rare genetic variants that occur in protein-coding exons specific to alternative isoforms (“alt-exons”), often in a tissue-specific manner, identifying missense mutations that are predicted to be conserved or structurally damaging. We focus on missense variants, as these represent a prevalent mechanism of genomic variation, effects of which remain challenging to interpret^17^. As a case study, we analyze fine-mapped GWAS variants associated with severe COVID-19^18^ and identify a common variant located in an alternative first exon of an unannotated, lung cell-specific isoform of *DPP9*, causing a leucine to proline substitution within an isoform-specific N-terminal region of DPP9, predicted to disrupt an alpha-helix. Our findings reveal a largely unexplored mechanism by which common and rare disease-associated variants can act through coding changes in tissue-specific alternative isoforms, frequently absent from reference annotations. For rare variants, this can improve clinical classification of variants in monogenic disease, and for common variants associated with complex traits, can improve biological insights.

## Results

### Mutations in alternative isoform-specific exons

**Fig. 1:**
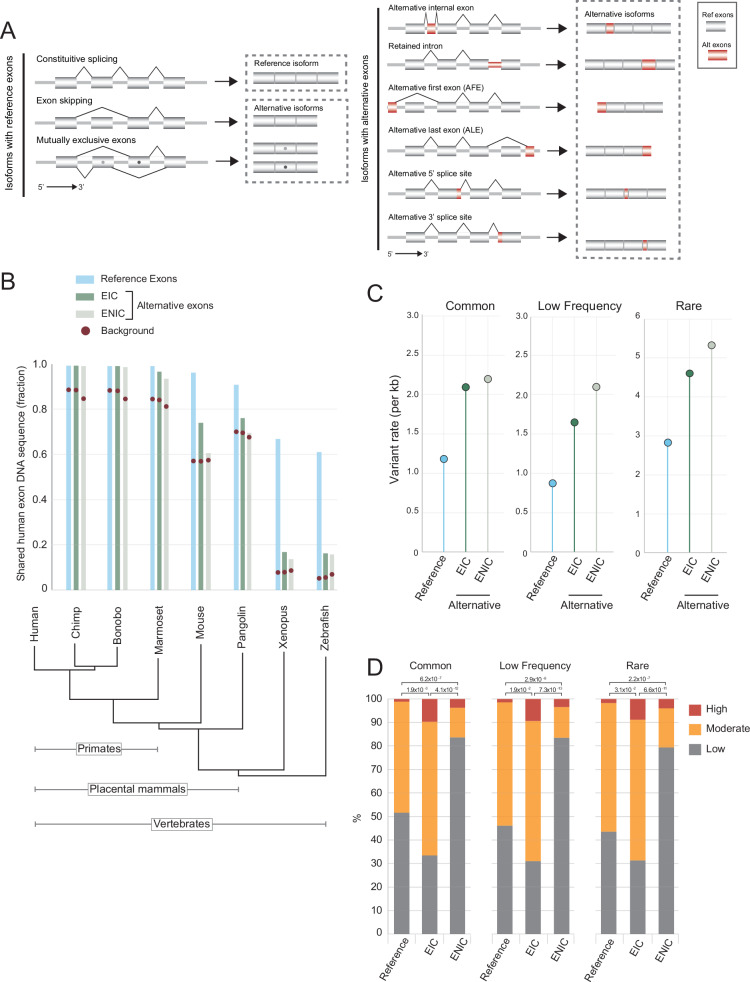
Features and classification of human exons. **A** Schematic of isoform structures, depicting ref-exons present in the reference isoform, and mechanisms generating alternative isoforms from ref-exons. Isoforms containing alt-exons (red), which are exons specific to alternative isoforms, can be formed through multiple mechanisms. **B** Evolutionary conservation of exons (ref-exon, EIC and ENIC) was determined by the fraction of exons per class shared between other species, including primates, placental mammals and evolutionarily distant vertebrates. Fraction of exons based on *n* = ~201 k exons for ref-exons, *n* = ~169 k for EIC exons, and *n* = ~182 k for ENIC exons. **C** Variant rate (per kilobase; kb) for each exon class was determined using single-nucleotide variants (SNV) from GnomAD (v4). Variants were grouped by minor allele frequency (MAF): common (≥0.05 to <0.5), low frequency (≥0.01 <0.05), and rare (≥0.001 and <0.01). The number of variants for each exon class was normalised by the combined exon genomic length to determine the per kb rate. Number of variants (*n*) for common variants are ~83 k for ref-exons, ~84 k for EIC, ~298 k for ENIC. For low frequency variants, *n* = ~61 k for ref-exons, ~65 k for EIC, ~284 k for ENIC, and for rare variants, *n* = ~196 k for ref-exons, ~182 k for EIC, ~718 k for ENIC. **D** Percentage bar chart of SNV impact on coding sequences using isoform-aware annotation. Variants were grouped by allele frequency from gnomAD. Predicted coding variant annotations were grouped into low (synonymous), moderate (missense), and high (frameshift, stop gain, stop lost and start gain). Where variants are annotated to multiple transcript isoforms, the most severe coding impact was considered. The total number of variants (*n*) for common variants are ~29 k for ref-exons, ~3.6 k for EIC, and ~1.85 M for ENIC. For low frequency variants *n* = ~22 k for ref-exon, ~ 2.8 k for EIC and ~1.7 M for ENIC. For rare variants *n* = ~92 k for ref-exons, ~10 k for EIC, and ~5.6 M for ENIC. *P*-values determined by one-way ANOVA. Source data are provided as a Source Data file.

To determine the contribution of genetic variants to coding mutations in alternative isoforms, we first classified exons by their relation to reference transcript isoforms. We considered transcripts from a meta-analysis of long-read RNA-sequencing (RNA-seq) based on GTEx, ENCODE and other datasets^19^, extracted exons, and classified these into exons present in reference transcript isoforms (ref-exons) or exons specific to alternative transcript isoforms (alt-exons). Reference transcript isoforms were defined using the Matched Annotation from NCBI and EMBL-EBI (MANE) Select transcript^20^. Although exons in the MANE Select transcripts can also appear in alternative isoforms, we classify all exons present in the MANE Select transcripts as ref-exons (Fig. 1A). Alt-exons were further classed into those annotated in Ensembl (exons in catalogue; EIC) or unannotated exons from long-read RNA-seq transcripts (exons not in catalogue; ENIC). Across the human genome, there were over 200 k ref-exons, while alt-exon counts were approximately 170 k for EIC and 180 k for ENIC (Supplementary Data 1). Relative to the reference transcript, we classified EIC into alternative: first exons (AFEs), internal exons, last exons, 5′ or 3′ splice sites, or single exons (Supplementary Data 2). For EIC, 5′ and 3′ splice site extensions were most common, followed by AFEs. We observe a medium of eight ref-exons per gene, 7 for EIC alt-exons, and 9 ENIC alt-exons per gene (Fig. S1A). Over 20% of genomic bases of ref-exons are in 5′ or 3′ untranslated regions (UTRs), compared to 3 and 1.7% of EIC and ENIC, respectively (Supplementary Data 3). ~59% of ref-exon genomic bases can be UTRs or coding, due to transcript isoform-specific start codon usage.

To consider evolutionary conservation as a mark of functional exons^21,22^, we performed a pairwise analysis of exon classes using sequence orthology (Fig. 1B). Ref-exons and alt-exons have a high fraction of orthologous exons in primates, but alt-exons were less conserved than ref-exons in placental and more distant vertebrate comparisons. Ref-exons have positive conservation scores, while alt-exons have near-neutral conservation scores (Fig. S1B). Ref-exons that are 5′UTR have a higher conservation score than those from Alt exons, but this is not the case for 3′UTRs (Fig. S1C). Some alt-exons may have evolved more recently, being primate- or human-specific. Transposable elements have been proposed to contribute to accelerated genomic regions^23^. Consistent with this, alt-exons have a higher fraction of exons harboring repetitive sequence, especially Short and Long Interspersed Nuclear elements (SINEs and LINES), compared to ref-exons (Fig. S1D). Within the SINE family, the primate-specific Alu elements, which have been implicated in exonization events^24^, were most abundant (Supplementary Data 4). Together, these data show that alt-exons have evolved recently, and many may have originated from retroelement transposition.

To understand the contribution of genetic variants to protein-coding regions, we analyzed the population prevalence of genetic variants in exons, using aggregated allelic frequencies from gnomAD^25^. We grouped single-nucleotide variants (SNVs) by their minor allele frequency (MAF) into common, low-frequency, and rare, observing comparable distribution of variants in exon classes (Fig. S1E). Across variant frequency groups, variant rate (variants per kilobase) for alt-exons was higher (1.7–2.4-fold) than for ref-exons (Fig. 1C). We next considered the impact of variants on protein-coding sequences, using isoform-aware variant annotation in Ensembl annotated isoforms. We grouped the variant impacts into low (synonymous), moderate (missense), and high (including frameshift, stop gain, stop loss and start gain), and took the highest severity for a variant where the variant occurs in multiple transcript isoforms (Fig. 1D). For common variants, the majority (51.6%) in ref-exons were synonymous, while in alt-exons they were missense mutations (56.8%). Across all MAF groups, common to rare, the proportion of variants causing moderate and high impact were significantly higher in alt-exons than ref-exons. Thus, alt-exons have a higher variant burden and a higher proportion of damaging variants.

### Disease-associated variants in tissue-specific alternative isoforms

Genetic variants associated with disease, whether from GWAS or rare variant databases, are rarely interpreted in the context of transcript isoforms^26^, even though many alternative isoforms show tissue-specific expression and can encode distinct proteins. Therefore, we investigated the extent to which disease-associated variants map to alt-exons and whether these events may have functional consequences.

**Fig. 2:**
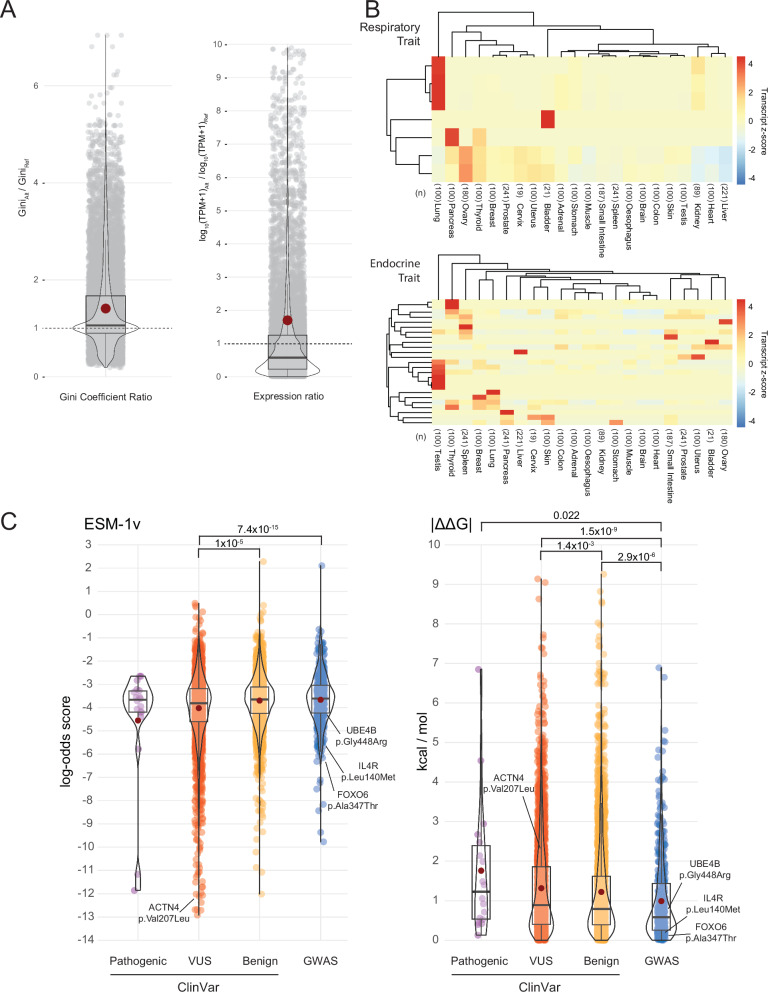
Common and rare variants are associated with tissue-specific isoforms. **A** Box and violin plots of alternative isoform to reference isoform ratios (*n* = ~132 K alternative transcripts) for tissue-specificity metric (Gini coefficient ratios, left), and transformed expression (log10(transcripts per million (TPM) + 1) ratios, right). GWAS variant-mapped alternative isoforms and gene-matched reference isoforms were annotated with Gini coefficients and TPM values from long-read RNA-seq of 22 GTEx tissues. For transformed TPM, ratios were determined from the highest expression in any GTEx tissue. Mean is shown in red. Central line is the median, with box showing first quartile and third quartile, and vertical lines show minimum and maximum. **B** Heatmap of alternative transcript isoform expression for isoforms associated with grouped GWAS variant traits. Expression is median of long-read RNA-seq transcripts from 22 GTEx tissues (*n* = number of biological replicates per tissue). Expression as z-score of log_10_TPM. Hierarchical clustering for tissues and variants was performed using the ward method. **C** Violin and box plots of evolutionary scale modelling (ESM)−1v and FoldX calculated protein thermodynamic stability difference in the Gibbs free energy (ΔΔG) scores. Predicted ClinVar (*n* = variants) and GWAS catalog (*n* = 490 variants) missense variant impact in variant-associated alternative isoforms were based on predicted Alphafold 3 (AF3) structures. ClinVar variants were grouped by ClinVar annotated pathogenicity into pathogenic (*n* = 18 variants), VUS (*n* = 2824 variants) and benign (*n* = 3436 variants). Mean is shown in red. Central line is the median, with box showing first quartile and third quartile, and vertical lines show minimum and maximum. *p*-values were calculated using Pairwise Wilcoxon Rank Sum Tests (two-sided) with Benjamini–Hochberg (BH) correction for multiple testing. Source data are provided as a Source Data file.

From the GWAS Catalog^27^, we find ~40,000 variants mapping to alt-exons (EIC) across ~15,000 alternative transcript isoforms from GTEx long-read RNA-seq data from 22 tissues. This compares to ~24,000 GWAS Catalog variants mapping to ref-exons. We prioritised mapping to EIC over ENIC, as transcripts that incorporate ENICs were also observed to have lower expression, and high rates of nonsense mediate decay (NMD) (Fig. S2A). Using Gini coefficients to quantify tissue specificity^28–30^, we found that most alternative isoforms showed more tissue-specific expression than their matched reference isoform (Fig. 2A). GWAS Catalog variants mapping to ref-exons showed higher conservation compared to those mapping to alt-exons (Fig. S2B). While alternative isoforms are typically expressed at lower levels, 32% exceeded the expression of the reference in at least one tissue (Fig. 2A), consistent with previous estimates^5^. Together, 20% of alternative transcripts had higher tissue-specificity and higher expression than the reference isoform (Fig. S2C). Grouping GWAS traits into biological systems, for example, clustering metabolic or immunological-associated traits, we find that GWAS variants are enriched in alt-exons across biological systems (Fig. S2D). The effect sizes (odds ratio) for these variants in ref-exon and alt-exons are similar (Fig. S3A). Variant-associated alternative transcript isoforms were highly expressed in relevant tissues. For example, variants associated with lung, endocrine, or hematological traits map to alternative isoforms that are expressed in diseases-relevant tissues (Figs. 2B and S3B). Additionally, mapping multi-ancestry fine-mapped variants to alt-exons using public data for 14 traits from the All of Us cohort^31^, and 94 traits from the UK BioBank cohort^32^, identified variants with high Posterior Inclusion Probability (PIP), and effect sizes (Fig. S3C). Fine-mapped All of US and UK BioBank variants were enriched in ref- and alt-exons, 1.4 versus 1.7-fold, respectively for All of Us, 2.1-fold for both ref- and alt-exons for UK BioBank variants. No significant difference in enrichment between ref- and alt-exons was observed.

Next, we sought to investigate the impacts of disease-associated variants in alternative isoforms, considering the GWAS Catalog variants, as well as pathogenic, uncertain significance and benign variants from ClinVar. As expected, Conservation scores of ClinVar variants were higher than GWAS Catalog variants (Fig. S3D). To identify coding variants affecting alternative protein isoforms, variants were annotated against isoform-specific EIC coordinates and mapped to coding transcripts derived from long-read RNA-seq data. Variants were annotated for isoform-aware effects, with missense mutations representing the largest group (Fig. S3E). Of the alternative transcript isoforms that harbor variants mapping to an alternative isoform-specific exon, ~80% were unannotated transcripts (Supplementary Data 5), reinforcing the importance of long-read transcript discovery. Similar to GWAS variants, alternative isoforms with mapped ClinVar variants showed high tissue-specificity, although most have lower expression than the matched reference isoform (Fig. S4A). We further considered only those variants that lead to missense changes in alternate exons, excluding those predicted to disrupt splicing and transcripts predicted to undergo NMD.

To estimate the functional consequences of each missense variant, we used ESM-1v^33^, a large protein language model that predicts the effect of amino acid substitutions based solely on sequence context, avoiding the necessity of deep sequence alignments required by most variant effect predictors, that limit applicability to alternate isoforms. To consider the impacts of missense variants on protein structure, we used AlphaFold 3 (AF3)^34^ to model the structures of ~17,000 alternative isoforms. We assessed the structural impact of each variant by estimating changes in Gibbs free energy of folding (ΔΔG) using FoldX^35^. We consider the absolute ΔΔG due to the observation that this better reflects pathogenicity^36^, likely because both stabilizing and destabilizing variants can cause disease. As expected, ClinVar pathogenic variants are the most damaging, with ΔΔG having better discrimination than ESM-1v (Fig. 2C). However, this group is small, with only 18 pathogenic variants mapping to missense changes in alternative isoforms. The low number likely reflects variants not annotated as coding in alternative isoforms often being overlooked in clinical interpretation. Despite filtering by SpliceAI scores, for three of the 18 variants, the underlying mechanism is likely to be through splicing, as exonic variants are known to contribute to splicing^37^. The next most damaging group comprises ClinVar variants of uncertain significance (VUS), consistent with the expectation that some of these will be pathogenic. ΔΔG values for VUS mapping to alt-exons fall within the range of those for pathogenic reference isoforms, but globally with lower severity (Fig. S4B). ClinVar benign and GWAS variants are predicted to have the mildest impacts. This may represent GWAS variants that are not causal due to linkage disequilibrium, or exert relatively modest effects compared to rare variants implicated in Mendelian disease.

We highlight several cases in which disease-associated variants map to tissue-specific alternative isoforms and are predicted to be damaging. We first considered GWAS variants that contribute to complex traits. A common GWAS variant associated with vitamin D levels^38^ maps to a disordered region of an unannotated *FOXO6* isoform (Fig. S5A) that is highly expressed in ovaries (Fig. S5B). A rare GWAS variant (MAF 1.23 × 10^−6^) linked to eosinophil counts^39^ falls within an unannotated *IL4R* transcript (Fig. S5C, D) that is expressed in adrenal and spleen (Fig. S5D). A rare variant associated with atrial fibrillation^40,41^ affects a known UBE4B isoform expressed in heart and muscle (Fig. S5E, F), consistent with previous observations^42^.

Considering rare variants associated with monogenic disease, among ClinVar VUS, we identify a missense variant in an alpha-helical region of an unannotated ACTN4 isoform (Fig. S5G) expressed in brain, kidney and testes (Fig. S5H). *ACTN4* variants are associated with focal segmental glomerulosclerosis^43^, suggesting possible relevance. We also find four VUS in an alternative exon of *EYA4* (Eyes Absent 4), with high sequence homology to a downstream ref-exon (Fig. S6A) and encoding a conserved protein tyrosine phosphatase domain^44^ (Fig. S6B). These exons undergo mutually exclusive splicing, and the homology has likely arisen from a duplication event^45^. The predicted reference and alternative isoforms have structural homology (Fig. S6C), and variant effect scores for the VUS in the alternative isoform suggest they have a damaging effect (Fig. S6D). Pathogenic ClinVar EYA4 variants are associated with cardiomyopathy and hearing loss, and the alternate isoform has tissue-restricted expression, highest in muscle but also expressed in heart (Fig. S6E). While the expression of this *EYA4* isoform is lower than the reference transcript in non-diseased tissue (Fig. S6E), the expression in disease states is not known. Details of these variants and the associated alternative isoforms coding mutations are detailed in Supplemental Data 6.

### A common disease-associated variant in an alternative DPP9 isoform

**Fig. 3:**
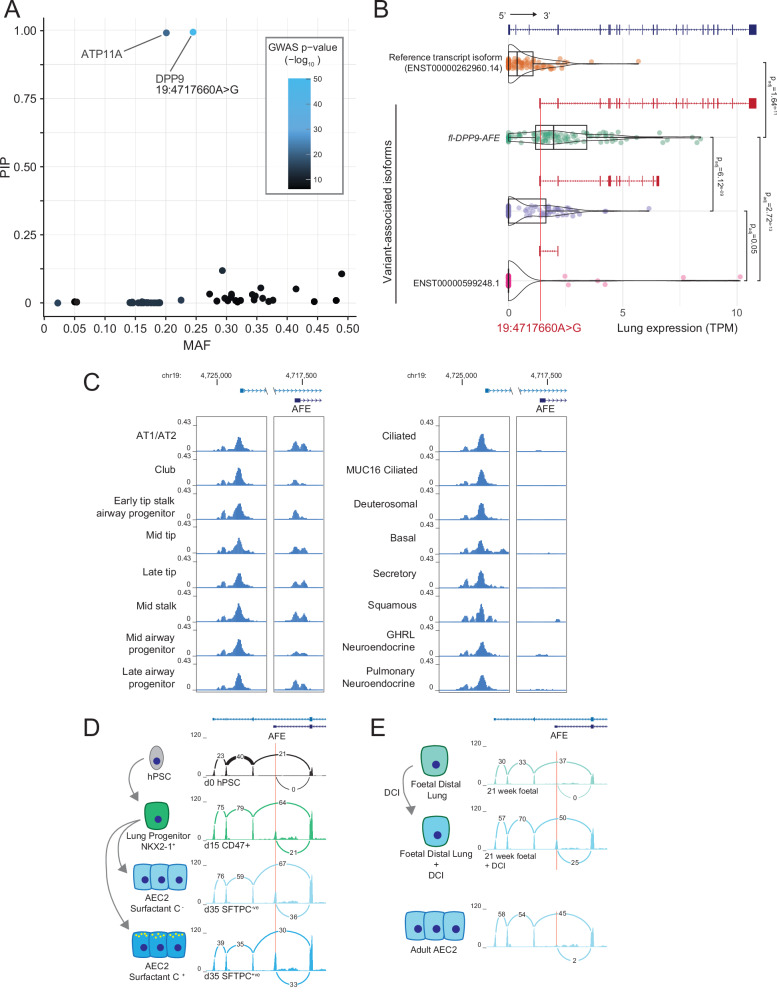
A variant-associated *DPP9* alternative isoform is expressed in lung. **A** Scatter plot of lead variants and 95% credible variant set from a meta-analysis of severe COVID-19 GWAS that intersect alt-exons in catalogue (EIC), plotting MAF from gnomAD against the Posterior Inclusion Probability (PIP). The GWAS *p*-value (−log_10_) is colour-coded in blue scale. **B** Violin and box plot for *DPP9* lung transcript expression (median transcript per million reads (TPM)) from long-read RNA-seq (GTEx; *n* = 100 lung tissue) for the reference transcript (blue), and transcript isoforms (red) intersecting 19:4717660A>G (rs12610495): full length *DPP9* alternative first exon (*fl-DPP9-AFE*) isoform, an unannotated *DPP9-AFE* isoform, and the annotated *DPP9-AFE* transcript (ENST00000599248.1 in Gencode V46). Central line is the median, with box showing first quartile and third quartile, and vertical lines show minimum and maximum. Pairwise Wilcoxon Rank Sum Tests (two-sided) with Benjamini–Hochberg (BH) correction for multiple testing. **C** Genome browser image of published single-cell ATAC-seq tracked for epithelial cell types from foetal human lung^59^. Pseudo-bulk ATAC-seq signal for the promoter of the reference transcript (left) and alternative promoter of the 19:4717660A>G variant-associated transcripts isoform (right). **D** Expression of *DPP9* splice junction reads of human pluripotent stem cells (hPSC) (grey) from published short-read RNA-seq^60^, differentiated into NKX2-1 positive lung progenitor cells (green), and surfactant C-positive and negative type-2 alveolar epithelial cells (AEC2) (blue). Tracks indicate normalised mapped reads from short-read RNA-seq, with numbers indicating normalised exon-exon spanning read counts, as mean of three biological replicates. **E** Expression of *DPP9* splice junction reads of foetal distal lung and adult AEC2 cells from published short-read RNA-seq^60^. Foetal distal lung cells were induced to differentiation using dexamethasone, cyclic AMP, and 3-isobutyl-1-methylxanthine (DCI). Source data are provided as a Source Data file.

To exemplify coding mutations in alternative isoforms associated with a specific phenotype, we used GWAS fine-mapping of severe COVID-19 variants^18^, consisting of 1848 variants at a posterior probability of 95%. We find 178 variants in coding regions, 98 of which occur in alt-exon EICs, 5.1-fold above the expected genomic distribution relative to the genomic size of the exons. This compares to 2.9-fold for ref-exon mapped variants (*p*-value 2.5 × 10^5^). The EIC variants in alt-exon have a range of MAFs, most of which (94/98) have a GWAS *p*-value of <1 × 10^−15^, but only two have a PIP > 0.99 (Fig. 3A). One common variant chr19:4717660-A > G (rs12610495, MAF = 0.29), with a PIP of 0.995 and a *p*-value of 9 × 10^−51^, is the lead variant at the *DPP9* locus^18^ and has previously been tagged in GWAS for lung fibrosis^46^. *DPP9* encodes a serine protease that cleaves N-terminal dipeptides^47,48^ and forms a complex with, and thereby suppresses, NLRP1—the primary inflammasome and viral sensor in barrier epithelial cells^49–51^. Loss or inhibition of DPP9 function leads to inflammasome activation and cell death by pyroptosis^51,52^. Rare coding variants in the reference isoform have been associated with inflammatory disease, including respiratory manifestations^53,54^. Co-localization analysis suggests chr19:4717660-A>G is shared in COVID-19 and lung fibrosis^55^ and has been previously suggested as an eQTL^55^ and sQTL^56^ for *DPP9*.

While the chr19:4717660-A>G variant has been frequently annotated as intronic to *DPP9*, we find it in an alt-exon. It intersects one GENCODE (v46) *DPP9* transcript (ENST00000599248.1), annotated as a short two-exon non-coding transcript, where the alt-exon forms an AFE. Using *DPP9* transcript isoforms from long-read RNA-seq^19^, we find three transcript isoforms that share the variant-harboring AFE. The lack of previous annotation of these transcripts is likely attributable to short-read sequencing being unable to resolve transcripts due to the shared 3′ exons. In GTEx long-read RNA-seq of lung, we observe high expression of one of the three AFE transcripts (Fig. 3B). A future GENCODE release will model the ENST00000599248 transcript with the coding sequence (CDS) extended to full length. Here, we refer to this full-length (fl) transcript as *fl-DPP9-AFE*. Compared to the reference transcript, *fl-DPP9-AFE* has higher expression in lung (Fig. 3B). It shares 19 exons with the reference transcript that encodes functional cytoplasmic DPP9 and only differs in the AFE, which forms an N-terminal extension. Using 22 tissues with GTEx long-read RNA-seq, we find 40 *DPP9* transcripts with detectable expression, with 33 unannotated in GENCODE. For these transcripts, we observe high tissue-specific transcript isoform expression, with *fl-DPP9-AFE* being specific to lung (Fig. S7A). However, not all of these 40 transcripts are likely to encode proteins with 1/40 predicted to be non-coding, and 18/40 predicted to undergo NMD (Fig. S7A).

We also identified the *fl-DPP9-AFE* isoform in published long-read RNA-seq datasets from a lung epithelial cell line, A549^57^. To independently validate *DPP9* transcript isoforms using deep targeted long-read RNA-seq, we developed Full-Length targEted capture using eXon probes for Isoform expRession (FLEXIR)-seq. This utilizes a custom panel of commercial exon probes, optimized for Oxford Nanopore Technologies (ONT) long-read sequencing of polyA transcripts (Fig. S7B), covering all exons of the reference *DPP9* isoform, plus a probe for the *DPP9* AFE. Probes were additionally designed for neighbouring genes to increase library complexity. FLEXIR-seq enriched for target genes, with off-target genes representing fewer overall reads (Fig. S8A), and associated with highly expressed genes (Fig. S8B). FLEXIR-seq replicates were highly correlated (Pearson coefficients 0.88–0.99) (Fig. S8C). High correlations were observed between some cell lines, likely due to the small number of genes in the exon panel. Relative expression of on-target genes showed good correlation with short-read total RNA-seq (*R*^2^ = 0.76), which we did not observe for off-target genes (*R*^2^ = 0.005) (Fig. S8D). Using this method, we confirmed the expression of *fl-DPP9-AFE* in lung epithelial cell lines A549, H358, HSAEC1-KT, HBEC3-KT and in N/TERT-1, a keratinocyte cell line (Fig. S8E). The *fl-DPP9-AFE* constituted 4–30% of total *DPP9* across cell lines. We further validated expression by qRT-PCR in A549, H358 and HSAEC1-KT cells, using exon-exon spanning primers that target the *DPP9-AFE* transcript (Fig. S9A).

As the chr19:4717660-A>G-variant-associated *fl-DPP9-AFE* transcript has lung-specific expression, we hypothesized that the AFE would utilize an alternative promoter, as previously observe for AFE isoforms^58^. Using ENCODE data from fetal lung, the promoter of the reference *DPP9* isoform shows accessible chromatin associated with a CpG island, and DNaseI footprints suggesting transcription factor occupancy (Fig. S9B). The 5′ regulatory element of *fl-DPP9-AFE* also showed open chromatin and DNaseI footprints, suggestive of an alternative promoter (Fig. S9B). In published single-cell ATAC-seq data from human fetal lung tissue^59^, we observe a peak at the *fl-DPP9-AFE* alternative promoter in a subset of lung epithelial cell types, from pseudo-bulk data (Fig. 3C), but not in mesenchyme, endothelial or immune cell types (Fig. S9C). The promoter of the reference transcript shows an ATAC peak in all cell types. To determine when during lung epithelial development *fl-DPP9-AFE* becomes expressed, we interrogated published short-read RNA-seq^60^ from lung epithelial differentiation of induced human pluripotent stem cells (hPSC). Taking exon spanning reads of the reference isoform and *fl-DPP9-AFE*, we detect no *fl-DPP9-AFE* expression in hPSC (Fig. 3D). *fl-DPP9-AFE* transcript expression emerged and increased during the differentiation into lung progenitor cells and lung epithelial cells, suggesting expression early in lung epithelial differentiation. There was minimal expression of *fl-DPP9-AFE* in quiescent fetal distal lung cells and adult alveolar epithelial type 2 (AT2) cells, but this increased in response to epithelial differentiation using dexamethasone, cyclic AMP, and 3-isobutyl-1-methyxanthine (DCI) in distal lung cells (Fig. 3E). These observations demonstrate that in lung, the *fl-DPP9-AFE* isoform is lung epithelial specific, with increased expression during lung epithelial differentiation.

### A DPP9 isoform-specific variant causes a missense mutation

**Fig. 4:**
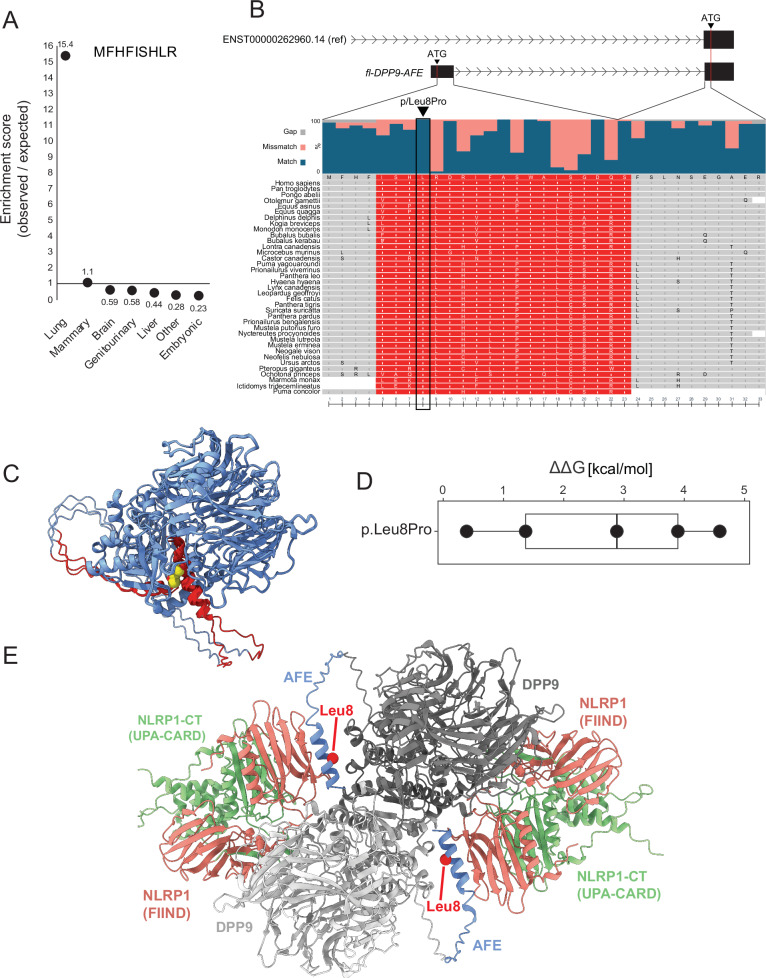
DPP9 common variant causes an isoform-specific N-terminus missense mutation. **A** Enrichment of the N-terminal peptide sequence encoded by the DPP9-AFE of the 19:4717660A>G-associated transcript isoform in proteomic meta-analysis. Enrichment score was calculated by the distribution of peptide-expressing tissues, over a background tissue distribution, a composite of the five highest expressed reference isoform peptides (Fig. S10A). **B** Multiple sequence alignment for amino acid conservation of the DPP9 alternate isoform AFE-encoding peptide using BLASTp. Red area is gapless across species, while grey indicates gap(s) in at least one species. **C** AlphaFold 3 (AF3) predicted structure of the DPP9 alternative isoform. DPP9 reference isoform in blue, N-terminus from the alternative isoform in red and p.Leu8Pro missense variant location in yellow. **D** Variant effect prediction of the p.Leu8Pro missense mutations using AF3 predicted structure and FoldX calculated protein thermodynamic stability measured as difference in the Gibbs free energy (ΔΔG). Boxplot showing the FoldX-predicted ΔΔG of p.Leu8Pro across five AF3 models. Central line is the median, with box showing first quartile and third quartile. **E** AF3 predicted ternary structure of the DPP9 alternative isoform-NLRP1 complex, consisting of a DPP9 dimer, and two NLRP1 molecules. DPP9 reference isoform in grey, DPP9 N-terminus of the alternative isoform in blue, NLRP1 FIIND (Function-to-Find domain) in orange, and NLRP1 C-terminus (CT), termed UPA-CARD (UNC5, PIDD, and Ankyrin-caspase recruitment domain), in green. Source data are provided as a Source Data file.

The *DPP9* reference isoform has two translation initiation sites^48^, the first generating an 892 amino acid (aa) DPP9-long isoform preferentially located in the nucleus through an N-terminal nuclear localization signal^61^, and the second generating an 863 aa cytosolic DPP9-short isoform^48,62^. DPP9-short specifically has been shown to inhibit NLRP1 inflammasome activation^63^. *fl-DPP9-AFE* shares all exons encoding the cytoplasmic DPP9-short isoform, but with a predicted 33 aa N-terminal extension translated from the chr19:4717660-A>G variant-harboring AFE. The AFE has an ATG start codon and is in-frame with downstream exons. Using a proteomic meta-analysis database^64^, we detect the *fl-DPP9-AFE* encoded N-terminal peptide, enriched in lung (Figs. 4A and S10A), consistent with observations from *fl-DPP9-AFE* transcript expression.

**Fig. 5:**
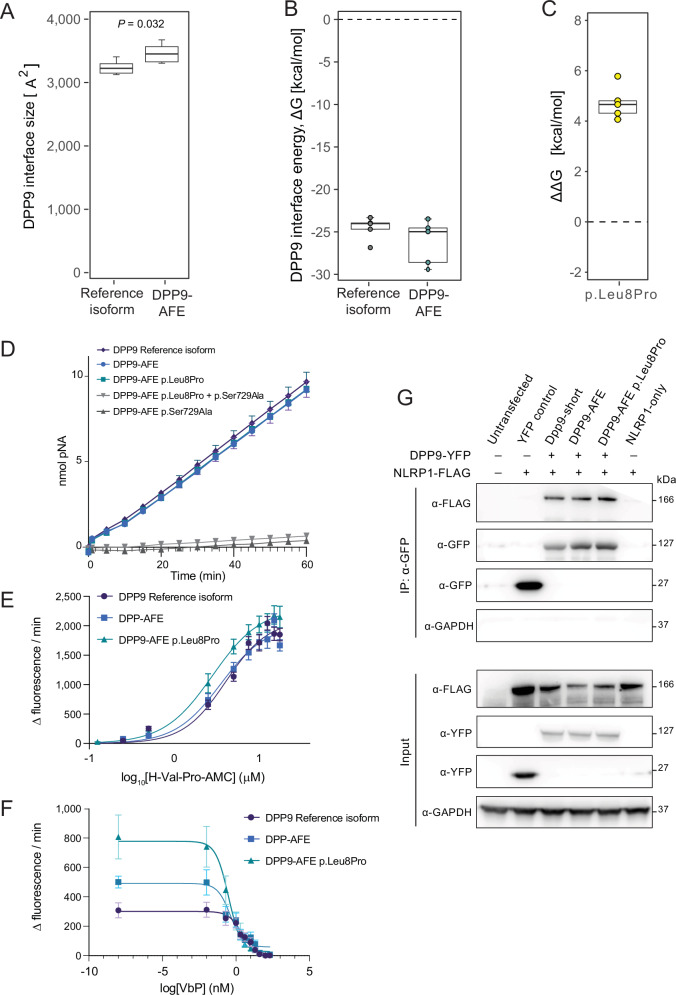
DPP9 missense mutation alters isoform activity. **A** AF3-predicted structure interface size between the N-terminus of the DPP9 alternative isoform and NLRP1 using FreeSASA, from five models. Central line is the median, with box showing first quartile and third quartile, and vertical lines show minimum and maximum, with *p*-value using one-way ANOVA. **B** AF3-predicted structure interaction energy between all chain pairs in the 2(NLRP1^full-length^–NLRP1^C-terminus^–DPP9) complex computed using FoldX 5.0 applied to the reference and alternative isoforms, using five models. Central line is the median, with box showing first quartile and third quartile. **C** FoldX estimations of ΔΔG from p.Leu8Pro missense mutations introduced into the N-terminus of the alternative DPP9 isoform of the AF3-predicted 2(NLRP1^full-length^–NLRP1^C-terminus^–DPP9) complex, using five models. Central line is the median, with box showing first quartile and third quartile. **D** DPP9-specific enzyme activity time course of DPP9 isoforms, with the p.Leu8Pro variant and, as a control, a catalytically dead (p.Ser729Ala) variant^96^, in whole cell lysates using H-Gly-Pro-pNA dipeptide substrate at saturating concentration (1 mM). Mean with error bars showing ± SEM. Values were derived from three biological replicates, measured in technical duplicate. **E** Enzyme kinetics for DPP9 reference and AFE isoforms, and the p.Leu8Pro variant form of DPP9-AFE, using H-Val-Pro-AMC dipeptide substrate. Fluorescence emission was detected upon cleavage of the fluorogenic salt 7-amino-4-methylcoumarin (AMC), with rate of hydrolysis fit to an allosteric sigmoidal model. Data points show mean ± SEM of six biological replicates. See Supplementary Data 7 for details. Mean values were derived from three biological replicates (*n* = 3). **F** As in (**E**) but for response to DPP inhibitor Val-boroPro (VbP). Mean with error bars showing ± SEM. Values were derived from six biological replicates (*n* = 6). **G** Western blot of co-immunoprecipitation for FLAG-tagged NLRP1 and YPF-tagged DPP9 reference and alternative isoforms, including the p.Leu8Pro mutation in the alternative isoform. Constructs were expressed in HEK293 cells, then immunoprecipitated with GFP antibodies to pull down YFP-tagged NLRP1. Images are presentative of three biological replicates. Source data are provided as a Source Data file.

The N-terminal DPP9 extension appears to have emerged within the Boreoeutheria magnaorder of placental mammals (Fig. S10B, C). In comparison, the DPP9 catalytic domain is highly conserved^65^ across the mammalian lineage, with orthologs also detected in the fungal kingdom (Fig. S11A). The A > G common variant, within the N-terminal extension, is predicted to cause a p.Leu8Pro missense mutation at a conserved site (Fig. 4B). Applying AF3 to fl-DPP9-AFE, the predicted structure resembles the recently solved cryo-electron microscopy structure of human DPP9-short^63^, but with the N-terminal extension forming an additional alpha-helix (Fig. 4C). FoldX suggests a helix-breaking impact of the leucine to proline substitution (Fig. 4D). The solved ternary DPP9-NLRP1 complex consists of two DPP9, each with two NLRP1 molecules—full-length NLRP1 and a C-terminal NLRP1 fragment^63^. Using AF3 to model the ternary complex with two DPP9 alternative isoform molecules (Fig. 4E), we find the N-terminal alpha-helix likely forms additional contacts with NLRP1 (Fig. 5A), increasing complex stability (Fig. 5B) compared to the reference isoform. Introducing p.Leu8Pro into the DPP9-AFE ternary complex with NLRP1 predicts disruption of these structural features (Fig. 5C).

DPP9 functions as a serine protease through a C-terminal α/β hydrolase domain. Assaying the enzymatic activity of the DPP9-AFE isoform and mutation effects, we find that DPP9-AFE has similar enzymatic activity to the reference DPP9-short protein in an in vitro H-Gly-Pro peptide hydrolysis assay^48^, and this is unaffected by the p.Leu8Pro mutation at saturating substrate concentrations (Fig. 5D). DPP9 can hydrolyze multiple proline-containing dipeptides. To explore substrate affinities, enzymatic kinetics were measured using substrates with differing charge and hydrophobicity. We find p.Leu8Pro DPP9-AFE to have a modestly higher affinity (lower K_half_) for H-Val-Pro compared with other DPP9 forms (Fig. 5E), but similar affinities for other dipeptides (Fig. S11B–D). DPP9-AFE has a higher affinity for the DPP inhibitor Val-boroPro than the reference DPP9^63^, and strikingly, this affinity is even further elevated in p.Leu8Pro DPP9-AFE (Fig. 5F). To validate the predicted structural interactions of the DPP9 isoforms with NLRP1, we co-immunoprecipitated YFP-tagged DPP9 isoforms and FLAG-tagged NLRP1 in HEK293T cells. Both the wild-type and p.Leu8Pro mutant DPP9-AFE isoforms showed interactions with NLRP1 comparable to those observed with DPP9-short (Fig. 5G), supporting the structural predictions.

## Discussion

Identifying the underlying mechanisms for both common and rare genetic variants remains a significant challenge. Variants impacting protein function are typically considered in relation to the reference isoform^66^. Here we show the prevalence of coding exons in alternative transcript isoforms that are not annotated in the reference human genome, and that are often highly tissue specific. While rare disease-causing coding variants in Mendelian disease are typically functionally damaging^67^, we demonstrate that some common variants in isoform-specific exons can cause damage to structures of isoform-specific protein regions. This highlights an underappreciated mechanism in variant interpretation—a discernible mutational effect for common and rare variants specific to alternative isoforms. Our framework can identify functional variants and prioritize functional alternative isoforms, distinguishing these from the broader background of transcript noise^68^. Expression in the relevant tissue, and relative to the reference isoform, should be taken into account when considering the contribution of alternative isoforms to pathomechanisms.

Our observation of alt-exons having a higher proportion of transposable elements, in particular Alu elements, is consistent with the evolution of novel human or primate-specific exons by transposable element-mediated exonization^69^. We observe a higher variant burden and greater proportion of missense mutations in alt-exons. The persistence of variants in alt-exons is likely through either recent evolutionary emergence or from weaker purifying selection compared to ref-exons, due to tissue-specific expression of the variant-harboring isoforms, or buffering by other functionally compensating isoforms.

Alternative isoforms can differ in molecular interactions and function^5,6,70^. We observe tissue-specific expression of variant-harboring alternative isoforms in disease-relevant tissues. We leveraged AF3-predicted structures to generate two variant effect scores and highlight various examples of likely structurally-damaging variants impacting isoform-specific protein regions. Inclusion of tissue-specific exons into protein structures is known to modify protein interactions^71^, thus coding variants in alt-exons could confer tissue-specific effects.

Accounting for alt-exons reclassifies ~19% of GWAS hits, although the majority remain non-coding (~74%). Amongst variants associated with severe COVID-19, we identified one within an unannotated, functional and lung epithelial-specific DPP9 isoform. The GWAS missense variant in this isoform is predicted to be structurally damaging, disrupting a predicted N-terminal alpha helix. DPP9 is the only known endogenous inhibitor of NLRP1, the primary inflammasome in barrier epithelial cells^49^. Upon detection of pathogen-associated danger signals, NLRP1 initiates an inflammatory response resulting in pro-inflammatory cytokine secretion and pyroptosis. SARS-CoV-2 infection has been shown to cause NLRP1 activation in lung epithelial cells^72^, consistent with the lung tissue type in which we find the variant-harboring DPP9 isoform expressed. The DPP9-NLRP1 axis is an important regulator of lung inflammation. Patients with rare loss-of-function mutations in DPP9 present with various immune disorders mediated by NLRP1 overactivation^53^, while an asthma-associated variant in NLRP1 (p.Met1184Val) has been reported to enhance binding of NLRP1 to DPP9^73,74^. The chr19:4717660-A>G *DPP9* variant has been reproducibly associated with both severe COVID-19 and lung fibrosis across multiple GWAS studies^18,46,75,76^, suggesting a unified mechanism of pulmonary inflammation and epithelial dysfunction. Functional enzymatic assessment of the DPP9-AFE p.Leu8Pro mutation shows altered affinity for ValPro dipeptides, suggesting favorable entrance of hydrophobic substrates into the catalytic pocket. This is consistent with the higher affinity of the structurally similar inhibitor, Val-boroPro, suggesting the DPP9-AFE isoform, with the common variant, is more sensitive to pharmacological inhibition.

Discrepancies between reference annotations and the diversity of alternative isoforms have been reported^77^ and has resulted in both false positive and negative clinical diagnosis of variants later re-annotated as coding variants in alternative isoforms^78^. Long-read sequencing of relevant cell types or tissues is required to avoid mis-annotation of transcripts, as demonstrated here for *DPP9*. We validated our findings by developing a full-length targeted capture RNA-seq method, FLEXIR-seq, to allow deep characterization and quantification of full-length polyA transcripts. FLEXIR-seq is analogous to similar methods^79^, but has been optimized for ONT long-read sequencing and exon probes, which have been reported to provide uniform coverage to preserve relative quantification^80^.

Our study has several limitations. First, we used consortia long-read RNA-seq atlases^3^ to determine tissue-specific expression and association with disease variation, but these datasets do not consider disease contexts. As disease-specific long-read transcriptomics emerge^81,82^, context-specific transcript isoform expression levels could improve identification of functional isoforms, variant interpretation and mechanistic insight^26^. Additionally, full-length transcriptomes have not been resolved at the single-cell level, thus our variant interpretation likely misses transcripts expressed in rare cell types. Second, for complex traits with common variant association, we used the GWAS catalog which has limitations in reported SNP-trait associations, either inherent to individual studies, such as sample sizes or ancestry, or where reported summary statistics likely miss causal variants in linkage disequilibrium. In this case, fine-mapped variants provide greater precision, and our analysis of a credible set of severe COVID-19 tagged variants suggests enrichment of variants in alt-exons. A similar approach for other complex traits may identify more variants that operate through coding mutations in alternative isoforms. Third, we focused on missense variants in our analysis of disease-associated variants and structure-based variant effect predictions. This represents the largest class of nonsynonymous coding mutations, and a challenging coding mutation class to interpret, compared to, for example, loss-of-function mutations^67^. However, some isoform-specific variants will cause other mutational coding effects such as frameshifts, which we have not considered here. Additionally, exonic variants identified as missense may alter splicing as their primary mechanism^37^, despite filtering by computational methods such as SpliceAI. Finally, we limited to variants in EICs when mapping disease-variants to alt-exons. We have not considered variants in ENICs—unannotated exons in unannotated transcripts uncovered by long-read RNA-seq^19^. These exons have low conservation, with transcripts lowly expressed and more likely to undergo NMD. Nevertheless, some of these novel exons, and their transcripts, may contribute to disease as previously suggested^83^. Further studies are required to validate and annotate these novel transcripts and their contribution to proteome diversity.

We anticipate our framework will have broad utility for the interpretation of functional variants for Mendelian diseases and complex traits, and to uncover mechanisms of common and rare variants. This could help clinical classification of rare variants in monogenic disease, and provide biological insight into complex traits for common variants. Assessment of both common and rare disease-associated variants in the context of isoform-specific effects, often through tissue-specific alternative isoforms, will help explain genetic contributions to human disease.

## Methods

### Defining exons classes

Long-read RNA-seq transcripts were obtained from a meta-analysis of long-read RNA-seq from human tissues^19^. Transcript models from ref. ^19^ were used. Transcripts associated with coding sequences were retained. Where indicated, transcripts expression and NMD predictions^19^ were used as further filter factors.

Exons were classed according to their intersection with the reference or alternative transcripts. The reference transcript was determined from the MANE annotation^20^, where exons present in the MANE transcript were classed as ref-exon. Exons present in alternative transcripts were classed as alt-exon and further divided into exons that were present in transcripts from Gencode V46, termed exons-in-catalog (EIC). Exons present in alternative transcripts, but not found in Gencode transcripts, were termed exons-not-in-catalog (ENIC). The alt-exons were further classed by position in the alternative transcript, into alternative first exons (AFE), alternative last exons, alternative internal exons (including retained intron), and 5′ or 3′ splice site extensions.

### Evolutionary conservation of exons

Conservation was determined by pairwise comparison between human and other species using UCSC Liftover with minMatch 0.5. The fraction of DNA sequences (exons) in synteny was determined by the number of sequences that match as a fraction of total queried sequences. A background model was determined using bedtools shuffle with the following options: -chrom -f 0 -noOverlapping, with repeat elements from UCSC excluded. Five background permutations of bedtools shuffle per DNA sequence set were determined and the mean fraction of syntenic sequences from the five permutations using LiftOver are shown.

Phylop scores were downloaded from the UCSC genome browser. The PhyloP score for exons was determined from the PhyloP470-way imputation. To determine the exon PhyloP score, the mean PhyloP was used from the per-nucleotide PhyloP score for each exon using bigWigAverageOverBed from the UCSC genome browser v1.04.00.

### Repeat elements intersection

Repeat elements were obtained from the RepeatMasker track from the UCSC genome browser. Repeat elements were grouped into classes as per UCSC into SINE, LINE, LTR, and DNA repeat elements. Where repeats did not fall into these groups, they were combined and labelled as “other”, which includes low complexity repeats, satellite repeats, and RNA repeats.

To determine the fraction of exons with repeat elements, exon coordinates were intersected with the RepeatMasker track using Bedtools Intersect.

### Exon class variant annotation

Variant occurrence rates were estimated using allele frequencies from gnomAD v4.1, considering only variants that passed quality control in genome and exome sequencing. Variants were binned by allele frequency as follows: common (≥0.05 to <0.5), low-frequency (≥0.01 to <0.05), and rare (≥0.001 to <0.01). For each exon class, the number of variants within each frequency bin was normalised per kilobase to account for differences in total base pair content across classes.

Molecular consequences of variants were annotated using Ensembl VEP version 112^84^, across all coding transcript isoforms available in Ensembl for each exon class. The analysis was limited to exons catalogued by Ensembl. Variant effects were grouped by the “Consequence” category assigned by Ensembl VEP into low (synonymous), moderate (missense), and high impact (stop loss, start loss, stop gain, frameshift). For ENIC variants, the full-length gene model from ref. ^19^ were used to determine low, moderate and high impact on coding sequences. In cases where a variant mapped to multiple transcripts, we considered the most severe consequence.

### SNP enrichment analysis

Background models to estimate enrichment was performed by sampling equal numbers of variants to the condition tested using variants from dbSNP 155 (*n* number of total variants). Background SNPs were intersected with features (e.g., alt-exons) and repeated with 10 permutations to obtain a mean background model for each tested condition. Enrichment scores were then computed as the number of observed SNPs (e.g., GWAS category) over the expected background mean over multiple permutations.

### DPP9 peptide analysis

To determine peptide expression from published mass spectroscopy data, DPP9 peptides from the reference isoform (long and short form) were identified using the Global Proteome Machine and Database^64^. The top five non-overlapping DPP9 peptides based on the peptide spectrum match of the Westmore-Standing plot for the long and short form DPP9 were used. Human cell line and tissue data for each protein sequence were amalgamated into tissue types. Tissue Enrichment score for the DPP9-AFE was calculated by using the proportion for each tissue mapped to the DPP9-AFE (observe), over the proportion of tissue associated with the combined tissues from shared (five highest) peptides (expected).

### Phylogenetic analysis

Phylogenetic trees were constructed from BLAST sequences (BLASTn for DNA sequence and BLASTp for protein sequence), allowing up to 5000 hits. DNA sequence identity ranged from 72 to 100%. Protein sequence identity ranged from 44 to 100%. Where BLAST sequence matches are find more than once for a given species, the highest percent identity was retained for phylogenetic tree generation. BLAST sequences underwent multiple alignment using ProbCons and alignment processing using Gblocks allowing least stringent alignments. Phylogenetic tree construction was performed by maximum likelihood using PhyML with SH-like approximate Likelihood-Ratio and the substitution model GTR for DNA and Dayhoff for protein. The circular phylogenetic tree was rendered using TreeDyn.

### Tissue-specific scoring

To determine tissue specificity of GTEx long-read RNA-seq, transcripts per million (TPM) counts were obtained from 22 tissues, with only transcripts with median expression > 0 in any tissue considered. To quantify tissue specificity of mRNA isoforms, the Gini coefficient, a nonparametric statistical measure of inequality, was computed for each transcript, where values close to 1.0 indicate high tissue specificity, while values near 0 reflect constitutive tissues expression. The Gini coefficient was computed using the ineq package R package v 0.2–13.

### Variant effect prediction in isoforms

To predict variant effects, variants from ClinVar (VCF weekly release 2024-08-05) and the GWAS Catalog were intersected with alt-exon EIC to limit the mapping to alternative isoform-specific exons. Genomic coordinates were first mapped with Ensembl VEP version 112^84,85^. Any variant with a non-synonymous consequence in either the ref-exon transcript or the UniProt primary isoform was discarded^86^. We also discarded variants with a predicted splice consequence in the ref-exon transcript or the primary isoform based on Ensembl VEP annotations, or with a masked SpliceAI score^87^ (sourced from Illumina BaseSpace Sequence Hub) of ≥ 0.2. Missense variants were further mapped to alternative transcript isoforms from FLIbase^19^ with a custom R script, based upon the DNA and protein sequences, the exon boundaries of the transcript, the DNA strand directionality, and the DNA sequence change for the variant. Transcripts predicted to undergo nonsense-mediated decay were excluded. Missense variant effects in the FLIbase-provided protein sequence for the isoforms were predicted with ESM-1v^33^. ESM-1v was run using the script^88^ available at https://github.com/facebookresearch/esm/blob/main/examples/variant-prediction/predict.py. Due to model limitations on sequence length, all sequences longer than 1020 residues were split into lengths of 1000 with a 50-residue overlap. Results within the overlapping window were averaged. The scoring strategy parameter used was “wt-marginals”. The models were run locally on an NVIDIA A100 GPU using Pytorch version 2.5.1 and Cuda toolkit version 12.4.

Protein sequences from FLIbase were also modelled with AlphaFold3^34^ using a3m formatted alignments generated with MMseqs2^89^ based on the UniRef and environmental sequence databases. Missense variant effects in the AlphaFold3 models were predicted with FoldX 5.0^35^ by first calling the RepairPDB command on the structure, followed by the BuildModel command to the wildtype model, and then estimate the change in Gibbs free energy of folding (ΔΔG) in the presence of the variant. The predicted Local Distance Difference Test for alternative isoforms mapping VUS and GWAs variants, and their matched reference isoforms, are shown in Fig. S12A. Predicted Aligned Error plots for structures used in Figs. 2, 4, S2, and S4 are shown in Fig. S12B.

### Cell culture

A549 (ATCC, CCL-185) and HEK293T (ATCC, CRL-3216) cells were maintained in DMEM (Life Technologies #41965039) supplemented with 10% fetal bovine serum (FBS) and 1% penicillin/streptomycin (P/S). H358 (ATCC, CRL-5807) and HCT116 (ATCC, CCL-247) cells were maintained in RPMI or McCoy’s 5A (Gibco # 26600023) media, respectively, each supplemented with 10% FBS 1% PS. HSAEC1-KT cells (ATCC, CRL-4050) were maintained in SABM BulletKit medium (Lonza CC-3119 and CC-4124) containing Bovine Pituitary Extract, hydrocortisone, human Epidermal Growth Factor, epinephrine, transferrin, insulin, retinoic acid, triiodothyronine and fatty acid-free BSA. N/TERT-1 keratinocytes (gift from Jim Rheinwald) were maintained in keratinocyte-SFM medium (Gibco # 17005042) supplemented with 2.5 µg human recombinant epidermal growth factor and 25 mg bovine pituitary extract from the same kit. All cell lines were grown at 37 °C and 5% CO_2_.

### Constructs and cloning

DPP9-short (Uniprot ID Q86TI2-1) and DPP9-AFE were amplified from H358 cDNA or in-house plasmid constructs using primers Short_HindIII_FW (GCGCAAGCTTATGGCCACCACCGGGACCCCAA) or AFE_HindIII_FW (GCGCAAGCTTATGTTTCATTTCATTTCTCATCT) and DPP9_SalI_RV (GCATGTCGACGAGGTATTCCTGTAGAAAGTGCA) by PCR (98 °C for 30 s, 30 cycles of 98 °C for 10 s, 69 °C for 20 s, 72 °C for 130 s and 72 °C for 3 min). Digested and purified products were subcloned into HindIII/SalI-digested pEYFP-N1 (BD Biosciences) to generate in-frame N-terminal YFP-tagged DPP9 isoform expression constructs. The risk variant rs12610495A>G, creating the DPP9-AFE L8 > P mutation, was introduced by PCR using primers L8P_FW (CAAAAATTCGATCCCGGGGATGAGAAATGAAATG) and L8P_RV (ATTTCATTTCTCATCCCCGGGATCGAATTTTTG) followed by DpnI template digestion. The catalytically inactive (S729A) mutation was introduced using S729A_FW (CCGTAGGCCCAGCCATGGATGGCAACTCG) and S729A_RV (TGGCTGGGCCTACGGGGGCTTCCTCTCG). The YFP-only control vector was made by excising the DPP9 insert using HindIII and AgeI restriction digestion and re-circularised by T4 ligation with a short linker fragment with compatible sites, made by annealing oligos linker_HindIII_FW (GAAGCTTGGTACCGCGGGCCCGGGATCC) and linker_AgeI_RV (GACCGGTGGATCCCGGGCCCGCGGTACC). All constructs were verified by Plasmidsaurus whole-plasmid sequencing.

### RNA extraction

Total RNA was isolated using the RNEasy Mini Kit from Qiagen (cat.no 74104) according to manufacturer’s instructions, including on column DNAse digestion.

### Quantitative RT-PCR

cDNA synthesis was performed using the SuperScriptTM First-Strand Synthesis System for RT-PCR from invitrogen (cat. no. 11904-018) with 1 μg RNA. qPCR was performed in 3 biological replicates with 3 technical replicates each using the LightCycler 480 SYBR Green I Master mix (Roche 04887352001) on the CFX96 Touch Real-Time PCR Detection System (Bio-Rad). The *C*_*t*_ values and primer efficiencies (PE) were determined by the CFX Manager software (Bio-Rad) based on a calibration 4-point serial dilution standard curve for each primer pair. The mean relative expression was calculated as PE^-Ct^ and normalized to the geomean of two reference genes, *GAPDH* and *SAFB*. *SAFB* was chosen as it is located on the same chromosome arm as *DPP9*, which has heterogeneous copy number in H358 cells. Primers used are listed in Supplemental Data 8.

### Full-Length targEted capture using eXon probes for IsofoRms (FLEXIR-seq)

The full FLEXIR-seq protocol can be found at protocols.io (dx.doi.org/10.17504/protocols.io.n2bvj9dnwlk5/v1) and uses commercially available kits and reagents. Briefly, long, stranded cDNA was prepared from 2 μg of total RNA using the ONT cDNA-PCR barcoding kit (SQK-PCS114.24), following manufacturer protocols. RNA underwent reverse transcription and strand-switching with Strand Switching Primer II to generate cDNA. 500 ng of cDNA was used for generation of long read libraries using the Twist Mechanical Fragmentation Library Preparation Kit (101280) and the Twist Universal Adapter System (101307). cDNA was subjected to end-repair and dA-tailing using ER/A enzyme, followed by Twist Universal Adaptor ligation (101307), and cleaned using DNA Purification Beads. Amplified indexed libraries were generated by adding Twist Unique Dual Index Primers to the adaptor ligated cDNA using KOD Xtreme hot start DNA polymerase (Sigma, (PN 71975-3)), then cleaned with DNA Purification Beads. Library fragments were quantified using DNA Broad range Qubit and fragment size was checked using Fragment analyser. Samples underwent targeted enrichment using Twist Standard Hyb and Wash Kit v2 (104445), Twist Universal Blockers (#100578), and a custom panel of exon probes. Amplified indexed libraries were pooled, then underwent probe hybridisation using the custom exon probe panel, Twist hybridisation mix, and universal blockers, incubated for 16 h at 70 °C. Dynabeads M-270 streptavidin beads (Invitrogen (65305)) in binding buffer were added to the hybridised library, and incubated at 68 °C, and immobilised using a magnetic stand, washed, and eluted. Supernatant underwent amplification by PCR and cleaned using DNA Purification Beads. The enriched library was validated and quantified using the Agilent bioanalyser with the High Sensitivity assay and a Qubit dsDNA High Sensitivity Assay. The enriched library underwent ONT adaptor ligation to prepare for long-read ONT sequencing using ONT Ligation Kit (V14, SQK-LSK114). Enriched libraries were end-repaired using NEBNext Ultra II end-prep enzyme, followed by adaptor ligation, then AMPure XP bead (Beckman Coulter Agencourt (A63881)) clean-up. Final library was quantified using the Qubit DNA high-sensitivity assay. Libraries were sequenced on the PromethION flow cell following manufacturer’s instructions.

FLEXIR-seq was applied to DPP9 isoforms using custom exon probes designed and synthesised by Twist Bioscience, including a probe to the AFE of the DPP9 isoform. To increase library complexity, probes targeting exons of neighbouring genes were included, targeting *MYDGF*, *TNFAIP8L1*, *FEM1A*, *SAFB*, for a total of 327 probes, using the Hg38 genome assembly. Probes have a length of 120 bp, with a mean GC% of 57%.

### Long-read RNA-sequencing

Target captured samples were multiplexed and sequenced across three flow cells on a PromentION platform (ONT) using the following software versions: ONT MinKNOW 25.03.7, bream .8.4.4, configuration 6.4.10 and ONT MinKNOW Core 6.4.8. Basecalling was performed using ONT Dorado v.7.8.3 in high-accuracy mode.

### FLEXIR-seq Read processing, demultiplexing and filtering

Reads passing the minimum Q9 score were demultiplexed based on i5 and i7 dual indexes using a custom Python script. Adapters were trimmed using cutadapt v.4.6^90^ with the parameters –report = minimal -m 100 –rc -e 0.12 -j 0 to search for adapters in both forward and reverse orientations, allowing a maximum error rate of 0.12 while filtering out reads shorter than 100 bp. Read quality was assessed prior to trimming using NanoPlot v.1.44.0 from the NanoPack2 package^91^. Nucleotide diversity at the read start and end were assessed NanoQC v.0.10.0^92^ both prior to and following adapter trimming to confirm successful trimming. Read quality was compared after and before adapter trimming using NanoComp v.1.24.0^93^.

### FLEXIR-seq alignment

Demultiplexed, trimmed reads were aligned to the GRCh38 human genome assembly using minimap2 v.2.28-r1209^91^ with the parameter -ax splice for splice junction aware mapping of cDNA reads, achieving >99.6% mapping across all samples. Sam files were converted to bam format, sorted and indexed using SAMtools v.1.20. Mapping statistics were generated using SAMtools flagstat command. Aligned bam files for each sample were visually inspected over the DPP9 region in the IGV genome browser.

### FLEXIR-seq gene and transcript quantification

Gene and transcript isoform counts were generated using IsoQuant v.3.7 with the parameters -d ont –report_novel_unspliced true –sqanti_output –genedb to enable de-novo isoform discovery optimised for Nanopore sequencing reads. The GRCh38 human reference genome assembly was provided as –reference and the GRCh38 human gene annotation in gtf format, additionally containing all DPP9 transcripts annotated in FLIbase^19^, provided as –genedb. Comparisons with short-read RNA-seq data used the following GEO Sequence Read Archive DataSets: A549 (SRR32141251, SRR32141252, SRR32141253); H358 (SRR24149739, SRR24149740, SRR24149741); HBEC3 (SRR32905582, SRR32905583, SRR32905584); HSAEC1-KT (SRR28773308, SRR28773309, SRR287733010); N/TERT-1 (SRR19142533, SRR19142536, SRR19142539). Short-read RNA-seq reads were aligned using bowtie2 v2.5.3 to GRCh38, and mapped reads converted to gene counts using featureCounts v2.1.1, then normalised using transcripts per million (TPM).

### DPP9 isoform cell transfection and protein extraction

A549 cells at 70% confluence (1.5 × 10^6^ cells) in 6-well plates were transfected with DPP9 constructs^48,62^ using Lipofectamine™ 3000 (Invitrogen, Carlsbad, CA; L3000015) following manufacturer’s instruction. Twenty-four hours post-transfection, cells were washed twice with PBS (Gibco, Paisley; #10010023) and then harvested using TrypLE (Gibco, #12605028). Briefly, cells were treated with 0.5 mL of TrypLE for 5 min at 37 °C, neutralised in 1 mL of complete media, and spun down at 300 × *g* for 7 min at room temperature, then the pellet was washed twice with ice-cold PBS.

For whole cell lysate, cell pellets were resuspended in Lysis Buffer (50 mM Tris-HCl, pH 7.6, 1% Triton ×-100, 10% glycerol, 2 mM dithiothreitol (DTT), 1 mM EDTA) and 1× protease inhibitor cocktail (Roche, Mannheim, #11836170001) for 5 min on ice and then spun down at 10,000 × *g* for 5 min. Supernatant containing soluble protein lysate was stored at −30 °C.

For nuclear and cytosolic fractions, cell pellets were first resuspended in swelling buffer (10 mM HEPES pH 7.6, 10 mM KCl, 1 mM DTT, 1 mM EDTA, 0.1% Triton ×-100 with protease inhibitors) and incubated on ice for 5 min to swell up. Then, the solution was resuspended several times to disrupt the cell membrane. Samples were spun down at 2000 × *g* for 5 min at 4 °C and supernatant containing the cytosolic fraction was collected. Glycerol was added to a final concentration of 10%. The nuclear pellet was washed in swelling buffer (excluding Triton ×-100) and then resuspended in 20 mM Tris-Cl, 420 mM NaCl, 1.5 mM MgCl_2_, 0.2 mM EDTA, 25% glycerol and protease inhibitors. Salt concentration was adjusted using 5 M NaCl to disrupt the nuclear membrane. Nuclear lysate was vortexed and then spun down at 10,000 × *g* for 10 min at 4 °C. Supernatant containing soluble nuclear proteins was collected and stored at −30 °C.

### DPP9 enzymatic and inhibition assays

Protein lysates from A549 cells (1.5 × 10^6^) were diluted 1:100 in triple distilled water and then quantified using Pierce MicroBCA assay (Thermo Scientific (23235)) following the manufacturer’s instructions. To measure DPP9 enzyme activity, two assays were employed. The substrate H-Gly-Pro-ρ-nitroanilide (H-Gly-Pro-pNA, Bachem (4025614)) is hydrolysed by DPP4, 8 and 9 into pNA. Each assay used 15 µg of protein of cell lysate and was performed at pH 7.8 and 37 °C. DPP9^S729A/S729A^ gene knock-in mouse embryonic fibroblast (GKI MEF) cells, which lack DPP9 catalytic activity^96^, were used as a negative control. The first assay used sitagliptin, a potent selective DPP4 inhibitor, to measure DPP8/9 activity^90^. Protein samples were incubated in 1 mM DTT, 1 µM sitagliptin in a total of 50 µL Tris-EDTA (TE, Sigma-Aldrich (T9285)) buffer on ice for 10 min. Absorbance at 405 nm (for pNA) and 570 nm (background) were then measured as time zero then 50 µL of 2 mM H-Gly-Pro-pNA was added. Absorbances were measured every 5 min for 1 h^90,94^.

The second assay used sitagliptin and compound 42 (Cpd42), a novel selective DPP9 inhibitor^91,95^, to measure DPP9 activity by subtraction. Background absorbance at 570 nm and blank (lysis buffer only) values were subtracted from absorbance at 405 nm. pNA product was extrapolated, using a pNA standard curve, as µmol pNA. To calculate DPP9 enzyme activity, pNA product from the 2nd assay was subtracted from the DPP8/9 measurement from the first assay. Enzyme activity was then converted to picomole pNA per min per µg of protein lysate.

Enzyme kinetics assays were performed using the following dipeptide substrates coupled to the fluorogenic cleaving group 7-amino-4-methylcoumarin (AMC): H-Valine-Proline (Enzo Life Sciences (BML-P448)), H-Tryptophan-Proline (Enzo (BML-P450)), H-Lysine-Proline (MedChemExpress (P4426A), and H-Aspartate-Proline (Mimotopes, customized). Substrates were dissolved in 100% DMSO as 2 mM stock solutions. A titration of cell lysate determined the quantity (2.5 µg protein) to use in the kinetics assay, in a range of substrate concentrations. To exclude DPP4 activity, 1 µM sitagliptin was added. Each assay included at least three technical replicates and three biological replicates. Values from blank controls were subtracted from the final readings. The rate of hydrolysis, ∆fluorescence/min, at each substrate concentration was calculated and plotted in GraphPad Prism V9.0. Vmax, K_half_ and Hill coefficient were calculated using allosteric sigmoidal equation.

For DPP9 inhibition, Val-boroPro (PT100/Talabostat/BXCL701), was used^97–100^. Val-boroPro was dissolved in 0.01 M HCl (pH 2.0) as a 30 µM stock and diluted to various working concentrations ranging from 0.05 nM to 1 µM using acidic water. Approximately 2.5 µg of protein from cell lysate per well, in duplicates, were incubated with Val-boroPro on ice for 5 min, with contained 1× Tris-EDTA buffer (pH 7.6) and 10 mM DTT. The final concentration of Val-boroPro was 0.01 to 200 nM. The DPP substrate, H-Val-Pro-AMC (Enzo, BML-P448), was used at a final concentration of 3 µM. Fluorescence (ex/em: 355/460 nm) was measured on a FLUOstar Omega (BMG LabTech, Germany) at 37 °C every 5 min. Values from blank controls (no protein) were subtracted from the final readings. ∆fluorescence/min for each substrate concentration was calculated and plotted in GraphPad Prism v9.0. To calculate IC_50_, Val-boroPro concentration was logarithmically transformed (value = 0 was replaced with 1 × 10^−^^9^), then curves were fitted using GraphPad Prism three-parameter dose-response curve.

$${{\rm{Y}}}={{\rm{Bottom}}}+({{\rm{Top}}}-{{\rm{Bottom}}})/\left(1+{{10}^{\Lambda}}^{({{X}-{{\rm{LogIC}}}}_{50})}\right)$$

IC_50_ for each isoform was reported with 95% confidence interval.

### Co-Immunoprecipitation assays and immunoblotting

HEK293T cells were maintained in DMEM (Life Technologies #41965039) supplemented with 10% FBS and 1% penicillin/streptomycin at 37 °C and 5% CO_2_. HEK293T cells were seeded at 1.2 × 106 cells/well in 6-well tissue culture plates and immediately transfected with plasmids encoding for DPP9-short-YFP, DPP9-AFE-YFP, DPP9-AFE(L8P)-YFP (each 1 µg) or YFP vector (0.65 µg) and NLRP1-Flag (1.37 µg) in equimolar ratios using PEI transfection reagent (Sigma Aldrich #919012) at a 3:1 ratio of PEI:DNA. 2 wells were transfected per condition. After 24 h, cells were harvested, washed 3 times with ice-cold PBS and lysed in 175 µl NP-40 lysis buffer (50 mM Tris-HCl (pH 7.4), 150 mM NaCl, 1% NP-40, 5 mM EDTA, Fisher Scientific #15403489) with one cOmplete protease inhibitor cocktail mini tablet (Roche #05892970001) per 20 ml and incubated on ice for 20 min followed by 10 cycles of 30 s on/off pulse sonication and centrifugation at 13,000 rpm for 10 min at 4 °C. Protein concentration was quantified using the Qubit protein assay (ThermoFisher #Q33212) and adjusted to 1.5 mg/200ul. Lysates were pre-cleared using 20 µl protein A dynabeads (Invitrogen # 10001D) for 20 min and incubated overnight with 25 µl ChromoTek GFP-Trap magnetic agarose beads (Proteintech #gtma) at 4 °C. After washing beads 4 × 7 min with NP-40 lysis buffer, bound proteins were eluted in 30 µl elution buffer (19.5 µl H2O, 7.5 µl 4× NuPAGE LDS sample loading buffer (Invitrogen #NP0007), 3 µl 10× NuPAGE sample reducing agent (Invitrogen #NP0009)) by boiling for 3 min at 95 °C. Immunoprecipitation samples and input samples (200 µg) were run in duplicate on NuPage 4–12% Bis-Tris gels (ThermoFisher #NP0323BOX) in 1× MOBS SDS running buffer (ThermoFisher #NP0001) at 150 V for 50 min. Membranes were transferred onto nitrocellulose membranes (ThermoFisher #IB301001) using an iBlot2 gel transfer device (ThermoFisher), blocked in 5% milk powder in TBS with 0.1% Tween-20 (TBS-T) for 1 h and incubated for 1 h at room temperature or overnight at 4 °C with primary antibody α-GFP (Proteintech #PABG1), α-Flag (Sigma #F1804) or α-GAPDH (Proteintech 10494-1-AP) in 5% milk powder in TBS-T. Membranes were washed 3 × 10 min in TBS-T, incubated with the appropriate horseradish peroxidase (HRP)-conjugated secondary antibody for 1 h and washed a further 3 × 10 min in TBS-T. Membranes were imaged with SuperSignal West Femto Maximum Sensitivity Substrate (ThermoFisher #34095) on the ImageQuant 800 imager (Cytiva).

The following antibodies were used: α-GFP, Proteintech #PABG1, 1:1000 dilution—Polyclonal (lot no: 70828032AB), α-Flag, Sigma #F1804, 1:750 dilution—Clone M2, (lot no: 0000308215), α-GAPDH, Proteintech 10494-1-AP, 1:1000 dilution—Polyclonal (lot no:N/A), Anti-rabbit IgG, HRP-linked Antibody, Cell Signalling, 7974S (lot no: 29), Anti mouse IgG, HRP linked Antibody, Cell Signalling, 7076S (lot no: 38).

### Reporting summary

Further information on research design is available in the Nature Portfolio Reporting Summary linked to this article.

## Supplementary information

Supplementary Information

Description of Additional Supplementary Files

Supplementary Data 1-8

Reporting Summary

Transparent Peer Review file

## Source data

Source Data

## Data Availability

FLEXIR-Seq data has been deposited in the Gene Expression Omnibus (GEO) repository under the accession number GSE303335. A web portal for gene or variant queries can be performed at https://genesis.igc.ed.ac.uk/. The exon class files, and variant annotations for ClinVar and GWAS variants mapping to alternative exons, with ESM-1v and Alphafold3-FoldX ΔΔG scores, are publicly available [https://github.com/sbiddie/Alternative_exons]. Alphafold3 structures for alternative isoforms have been deposited in the OSF repository [https://osf.io/btp73]. Source data are provided with this paper.
