# Supplementary material for: Disease-associated genetic variants can cause missense effects in tissue-specific protein isoforms
Source: Nat Commun. 2026 Jun 16;17:7627. doi: 10.1038/s41467-026-74280-w (PMC13429693; doi:10.1038/s41467-026-74280-w)
Supplement: Supplementary file 1 — Supplementary Information [file 41467_2026_74280_MOESM1_ESM.pdf]

Supplementary Figures

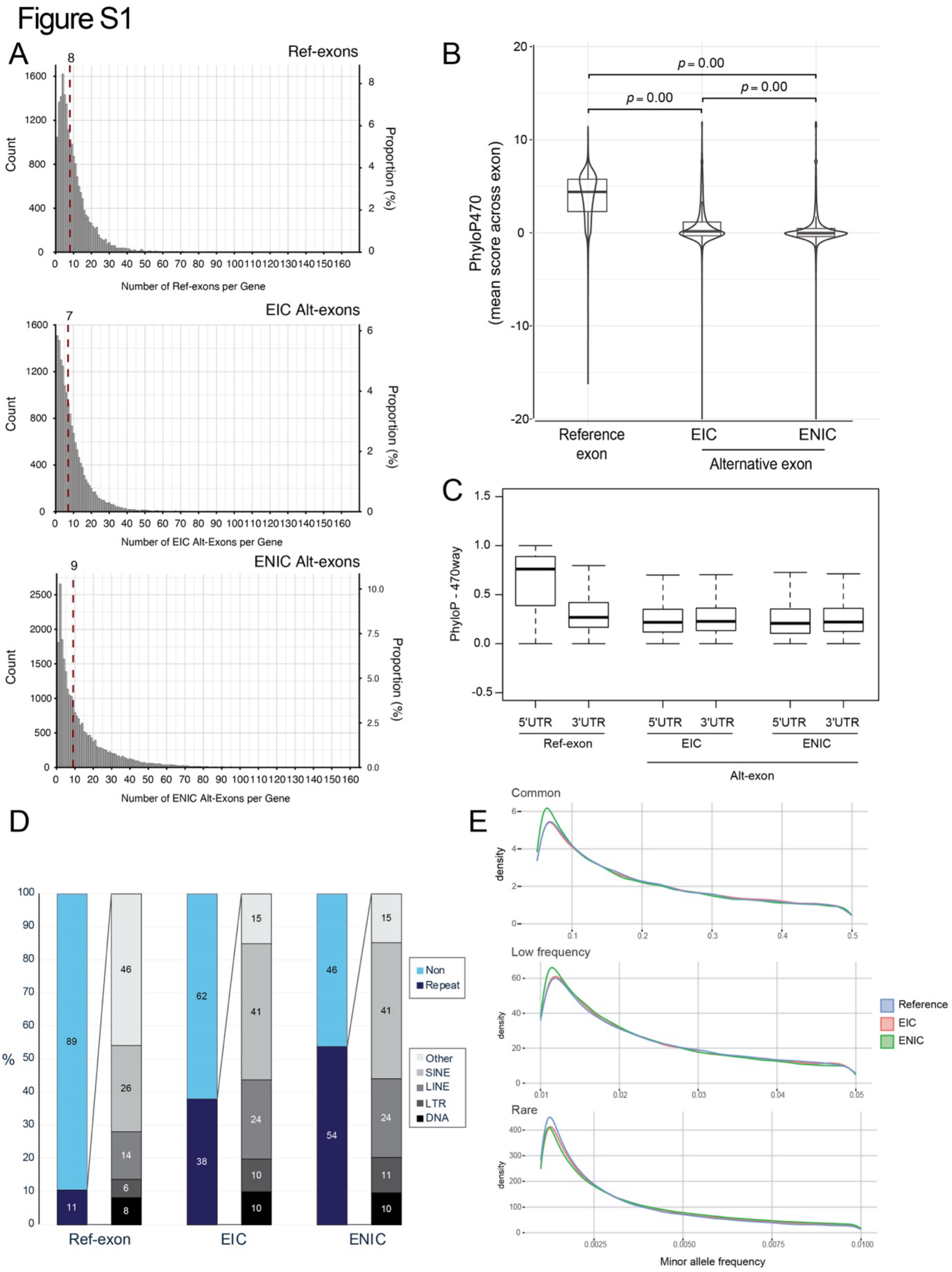

Figure S1. Characteristics of ref-exon and alt-exon classes

A. Histogram of exon counts per gene, for any transcript, by exon class - Reference exon (Ref-exon, n = ~19k genes), EIC (n = ~16k genes), or ENIC (n = ~25k genes), with median number per class shown.
B. Violin and boxplot of conservation score of exon classes using the mean 470-way PhyloP score across each exon. For ref-exons n = ~201k exons, for EIC n = ~169k exons, and for ENIC n = ~182k exons). Adjusted p-values are shown using the Howell-Game post-hoc test.
C. Boxplot of conservation scores of 5'UTRs and 3'UTRs grouped by exon class, using mean 470-way PhyloP scores. Number (n) of UTRs for ref-exons are for 5' n = ~86k, 3' n = ~11k, for EIC 5' n = ~4.7k, for 3' n = ~5.3k, for ENIC 5' n = ~6.9k, and 3' n = ~7.1k.
D. Proportional bar chart of repeat element types for the exons per class that intersect repetitive sequences. The number (n) of exons with repeat elements are n = ~21k for ref-exons, n = ~42k for EIC, and n = ~98k for ENIC.
E. Density distribution plots of single nucleotide variant (SNV) counts from gnomAD by exon class, across the allelic frequencies for minor allele frequency groups. For common variants n = ~23k variants, for EIC n = ~7.6k variants, for ENIC n = ~19k variants.
Source data are provided as a Source Data file.

Figure S2

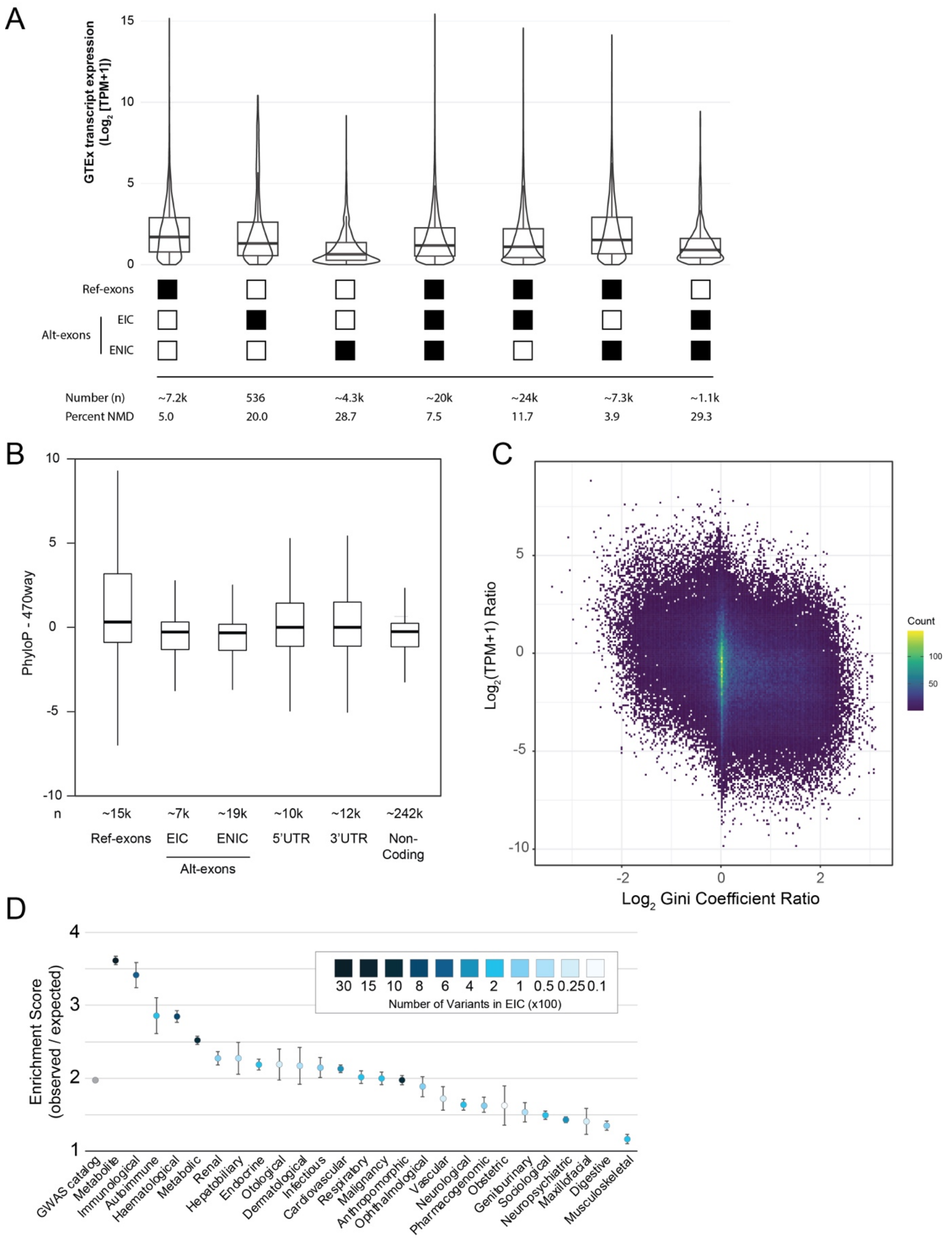

Figure S2. Expression and conservation of ref-exon and alt-exon classes
A. Violin and boxplots of expression of transcript isoforms from long-read RNA-seq (GTEx) grouped by composition of exon classes into ref-exons, EIC and ENIC exons, or combinations. The percent of transcripts predicted to undergo nonsense

mediated decay (NMD) are shown. The number of transcripts (n) are shown per transcript composition.
B. Boxplot of PhyloP-470way conservation scores for GWAS catalog variants mapped to genomic features. Number of variants per group indicated as n.
C. Scatter plot of of alternative transcript to reference isoforms ratios for expression (normalised TPM) against Gin coefficient, for n = ~11k transcripts isoforms.
D. Enrichment score for GWAS catalog variants in non-canonical exons in catalogue. Grouped GWAS catalog variants associated with non-canonical exons in catalogue (observed), with scores computed from ten imputations of randomly selected genomic variants (expected). The number of variants (n x100) are show as in blue scale.
Source data are provided as a Source Data file.

Figure S3

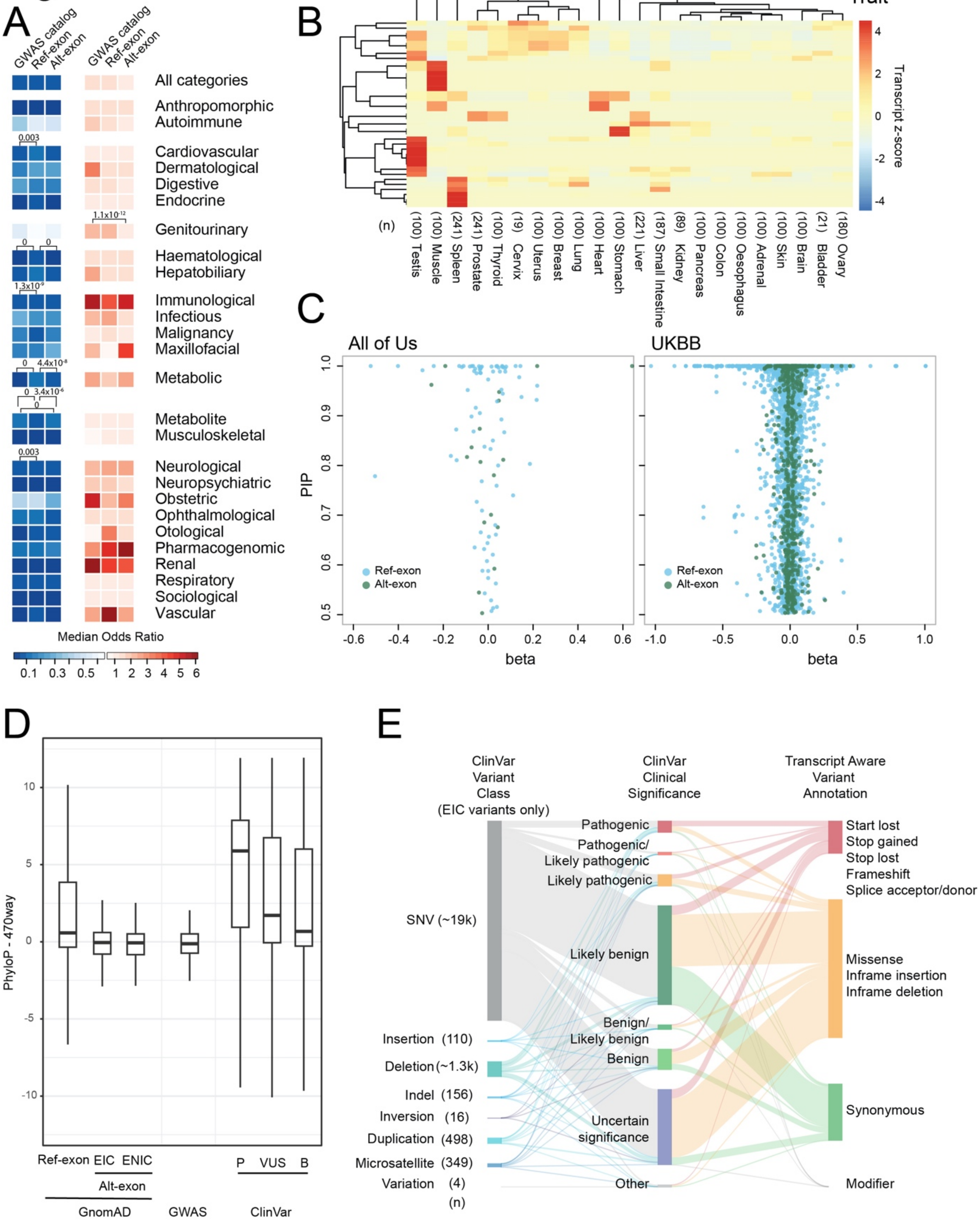

Figure S3. Common and rare variant association with alternative transcript isoforms.
A. Risk (red scale) and protective (blue scale) median odds ratio for GWAS catalog variants, mapping to reference or alternative exons, grouped by trait categories. P-value determined by the Game-Howell post-hoc method. Number of variants per group are provided in the source data file.
B. Heatmap of alternative transcript isoform expression for isoforms associated with variants from haematological GWAS traits. Expression is from long-read RNA-seq

transcripts in 22 GTEx tissues, with n = number of biological samples per tissue. Expression as z-score of log10 TPM. Hierarchical clustering for tissues and variants was performed using the ward method.
C. Scatter plot of the posterior inclusion probability (PIP), against the beta score, for fine-mapped variants for 14 traits from All of Us [32] (n = ~1.8k variants in ref-exons, and n = ~1.2k variants in EIC alt-exons), and 94 traits from UK biobank (UKBB) [31], n = ~177k variants in ref-exons, and n = ~130k variants in EIC alt-exons.
D. Boxplot of PhyloP-470way conservation scores for gnomAD variants mapped to exon classes, GWAS catalog variants, and ClinVar variants grouped into pathogenic, VUS and benign.
E. Proportional Sankey plot of ClinVar variants associated with non-canonical exons in catalogue by variant class, ClinVar annotated significance, and transcript isoform-aware variant annotation. n = number of variants per ClinVar class.
Source data are provided as a Source Data file.

Figure S4

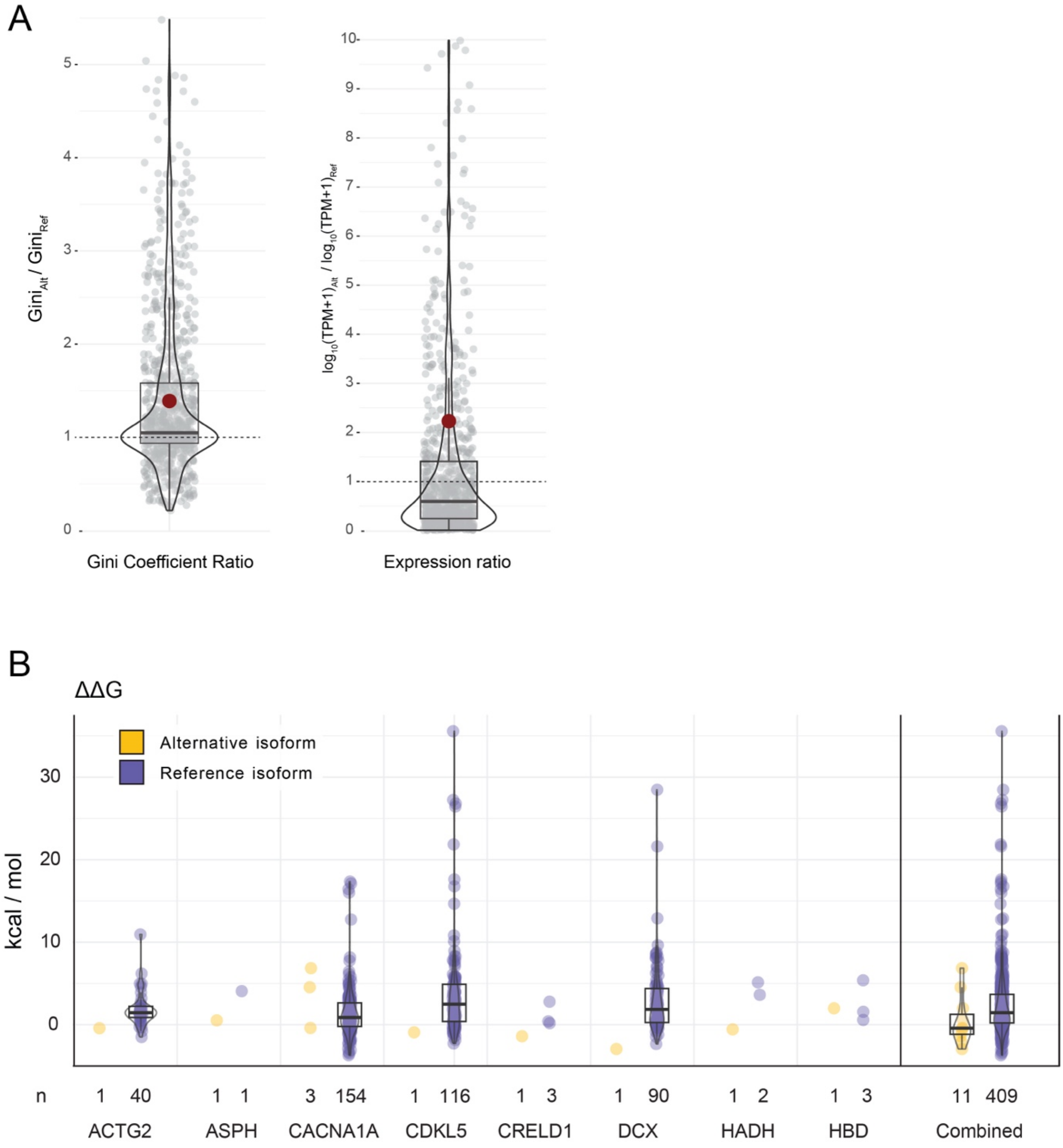

Figure S4. Characteristics of rare variant-associated alternative isoforms.
A. Box and violin plots of alternative isoform to reference isoform ratios for tissue-specificity metric (Gini coefficient ratios, left), and transformed expression ($\log_{10}$(transcripts per million (TPM)+1) ratios, right). Benign, likely benign, and variants of uncertain significance (VUS) ClinVar variant-mapping alternative isoforms and gene-matched reference isoforms were annotated with Gini coefficients and TPM values from long-read RNA-seq of 22 GTEx tissues. For transformed TPM, ratios were determined from the highest expression in any GTEx tissue. Where a ClinVar variant maps to multiple transcripts, only the alternative transcript isoform

with the most damaging variant effect score was used. n = 849 transcript isoforms. Mean is shown in red.
B. FoldX-derived Gibbs free energy (ΔΔG) values for alternative exon-mapping ClinVar VUS using alterative isoform AF3 predicted structures, and pathogenic ClinVar variants using reference isoform AF3 predicted structures, for eight genes, and their combined scores. Boxplots are shown where there are >3 variants. n = number of variants per isoform group.
Source data are provided as a Source Data file.

Figure S5

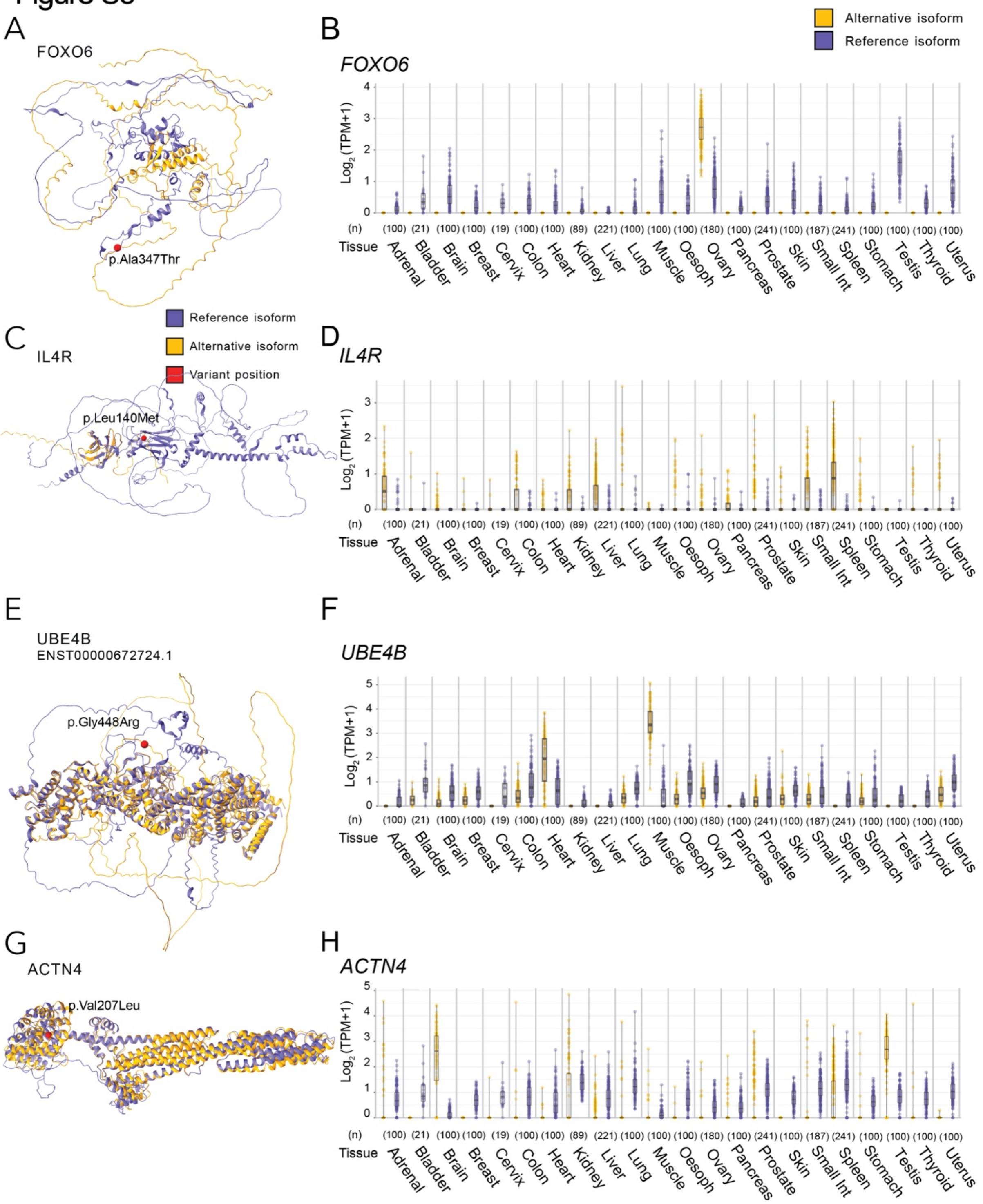

Figure S5. Predicted structures and tissue expression of isoforms.
A - B. Predicted AF3 structures for FOXO6 reference isoform (blue) and variant-associated alternative isoform (yellow) (A), and boxplots for alternative and reference transcript isoforms for *FOXO6* expression across 22 GTEx tissues from long-read RNA-seq (B), with n = number of biological replicates per tissue.
C - D. Predicted AF3 structures for IL4R reference isoform (blue) and variant-associated alternative isoform (yellow) (C), and boxplots for alternative and

reference transcript isoforms for *IL4R* expression across 22 GTEx tissues from long-read RNA-seq (D).
E - F. Predicted AF3 structures for UBE4B reference isoform (blue) and variant-associated alternative isoform (yellow) (E), and boxplots for alternative and reference transcript isoforms for *UBE4B* expression across 22 GTEx tissues from long-read RNA-seq (F).
G - H. Predicted AF3 structures for ACTN4 reference isoform (blue) and variant-associated alternative isoform (yellow) (G), and boxplots for alternative and reference transcript isoforms for *ACTN4* expression across 22 GTEx tissues from long-read RNA-seq (H).
Source data are provided as a Source Data file.

Figure S6

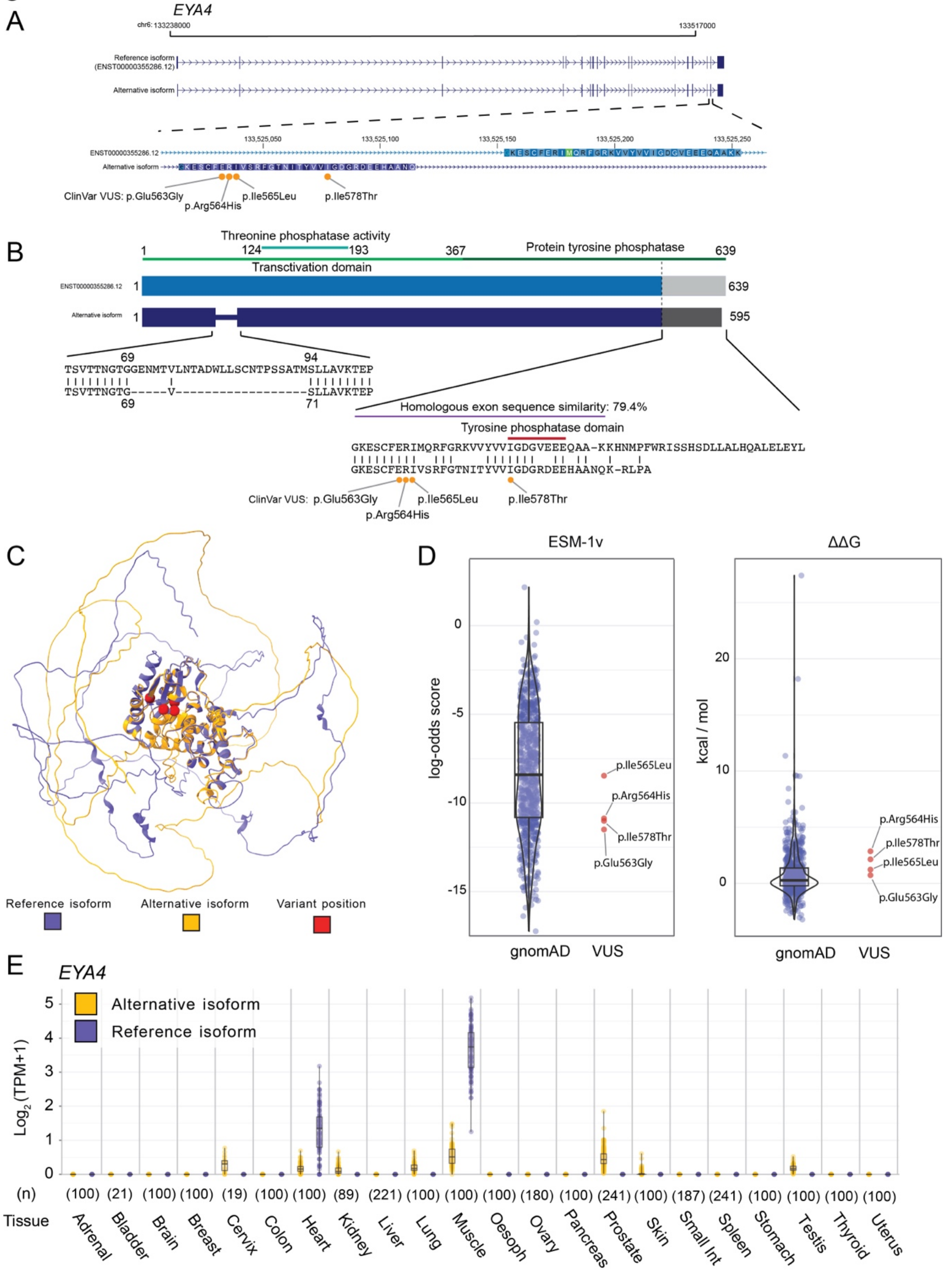

Figure S6. Variants of uncertain significance in an alternative homologous exon of an EYA4 isoform.

A. *EYA* transcript isoforms, showing the reference isoform (ENST00000355286.12), and an unannotated alternative isoform. The 3' end of the *EYA4* gene has two adjacent homologous exons that show mutually exclusive splicing. Four ClinVar

missense variants of uncertain significance (VUS) map to the alt-exon in the alternative transcript isoform.
B. The EYA4 isoform protein contains multiple functional domains, with shared isoform protein sequences, including the peptide sequence encoded by the homologous exons.
C. Predicted AF3 structure for the reference (purple) and alternative (yellow) EYA4 isoforms, with the ClinVar VUS indicated in red.
D. Variant effect prediction for four missense VUS (n = 4 variants) in the alternative EYA4 isoform using evolutionary scale modelling (ESM)-1v, and FoldX-derived thermodynamic stability difference in the Gibbs free energy (ΔΔG) ranked scores. Score for gnomAD variants (n = 721 variants) that map to the alternative isoform are shown for reference.
E. Boxplots for alternative and reference transcript isoforms for *EYA4* expression across 22 GTEx tissues from long-read RNA-seq. n = number of biological replicates per tissue.
Source data are provided as a Source Data file.

Figure S7

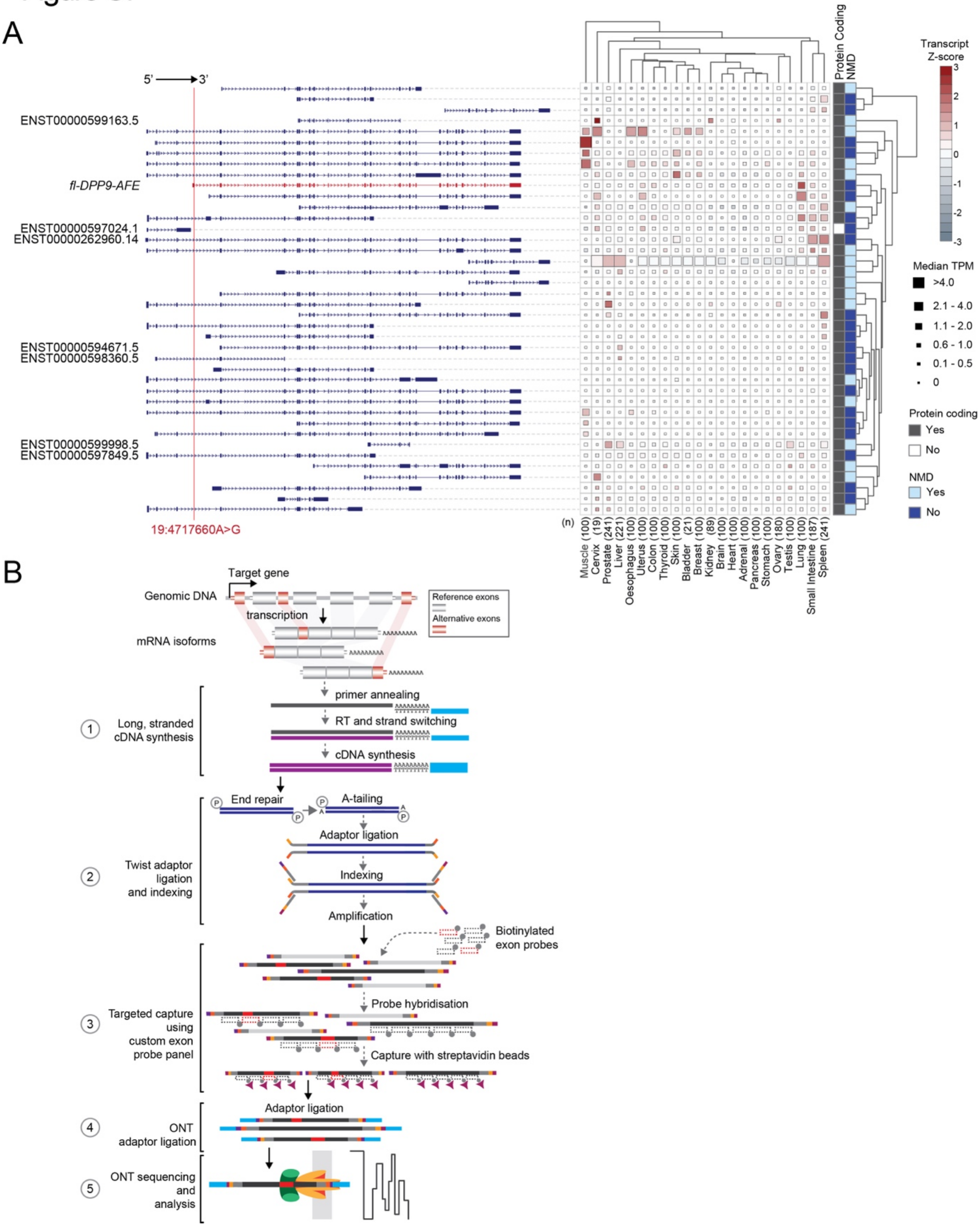

Figure S7. A DPP9 transcript expression, and targeted capture long-read RNA-seq protocol.

A. Heatmap of median *DPP9* transcript isoforms expression for 22 tissues from GTEx long-read RNA-seq (n = number of biological samples per tissue). Only transcripts with median expression > 0 for any tissue are shown. Z-scores are depicted per transcript, calculated across tissues. TPM, predicted protein coding and

nonsense-mediated decay (NMD) are shown. Hierarchical clustering of transcript and tissues used the ward method.
B. Schematic for FLEXIR-seq protocol, using a custom panel of biotinylated exon probes to capture full-length cDNA for long-read RNA sequencing.
Source data are provided as a Source Data file.

Figure S8

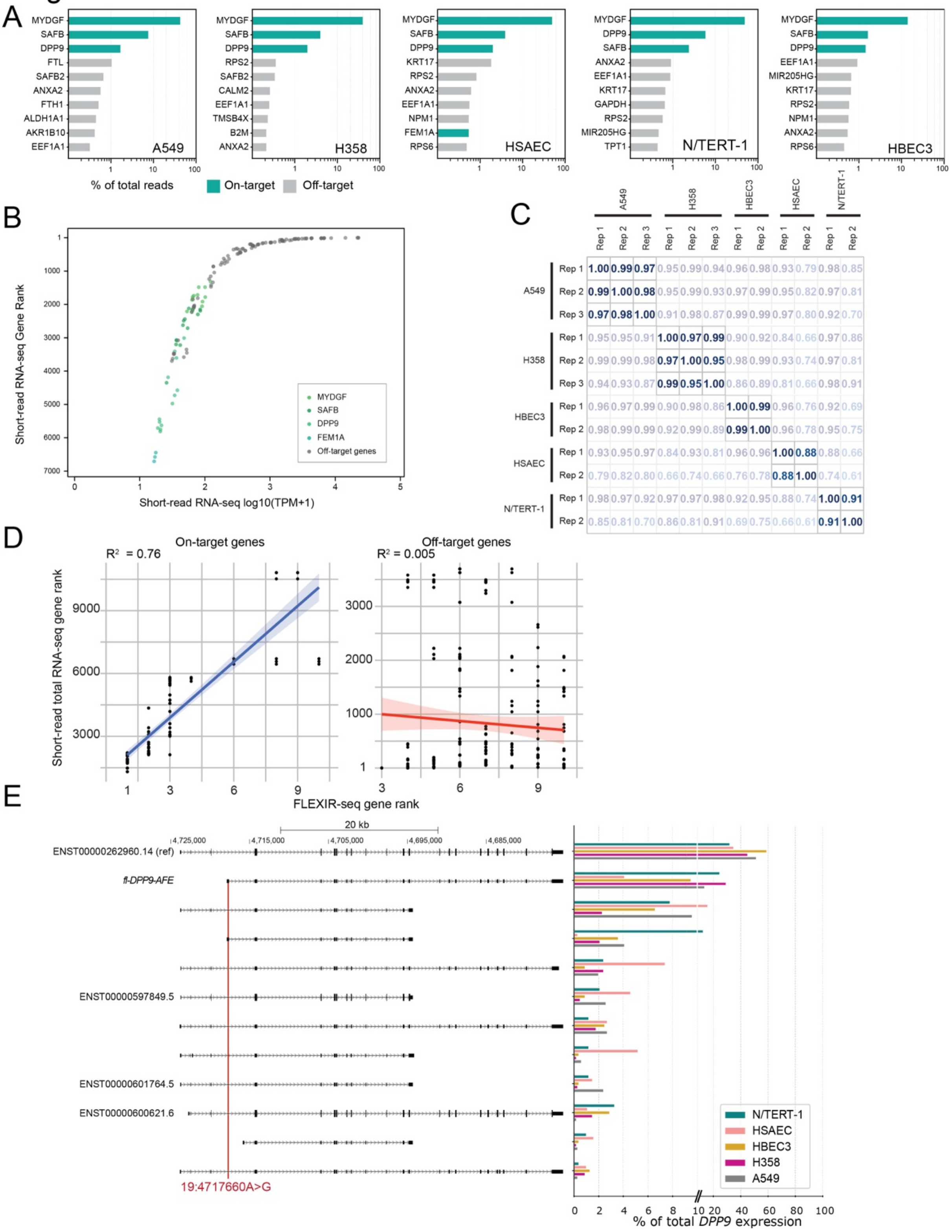

Figure S8. Full-Length targEted capture using eXon probes for IsofoRms (FLEXIR-seq)

A. Top ten ranking genes from FLEXIR-seq gene panel in five cell lines with pooled replicates, with on- and off-target genes based on targeted exon probe design. n = three biological replicates for A549 and H358, and two biological replicates for

HBEC3, HSAEC and N/TERT-1 cells. Transcript isoforms for each gene were combined per gene and calculated as percentage of total sequenced reads.
B. Scatter plot of short-read total RNA-seq gene ranks against transcripts per million (TPM, normalised as $\log_{10}$(TPM+1) for on-target and off-target genes identified using top 10 genes from FLEXIR-seq. Short-read total RNA-seq data were from three replicates for each cell line (n = 3).
C. Pearson coefficients for biological replicates of cell lines, using correlations of full-length transcripts for *DPP9* expressed as percentage of total reads for each replicate (n = 3 for A549 and H358, n = 2 for HBEC3, HSAEC and N/TERT-1 cells).
D. Linear model fit of the top 10 genes from FLEXIR-seq using gene ranks from replicates against gene ranks from short-read total RNA-seq for three replicates for each cell line. Linear model fits were generated separately for on-target genes (left and off-target genes (right). n = 3 for short-read and for long-read n = 3 for A549 and H358, n = 2 for HBEC3, HSAEC and N/TERT-1 cells.
E. *DPP9* transcript isoforms from targeted long-read RNA-seq (FLEXIR-seq) in five cell lines. n = 3 for short-read and for long-read n = 3 for A549 and H358, n = 2 for HBEC3, HSAEC and N/TERT-1 cells. Transcript expression is shown as percentage of total *DPP9* expression.
Source data are provided as a Source Data file.

Figure S9

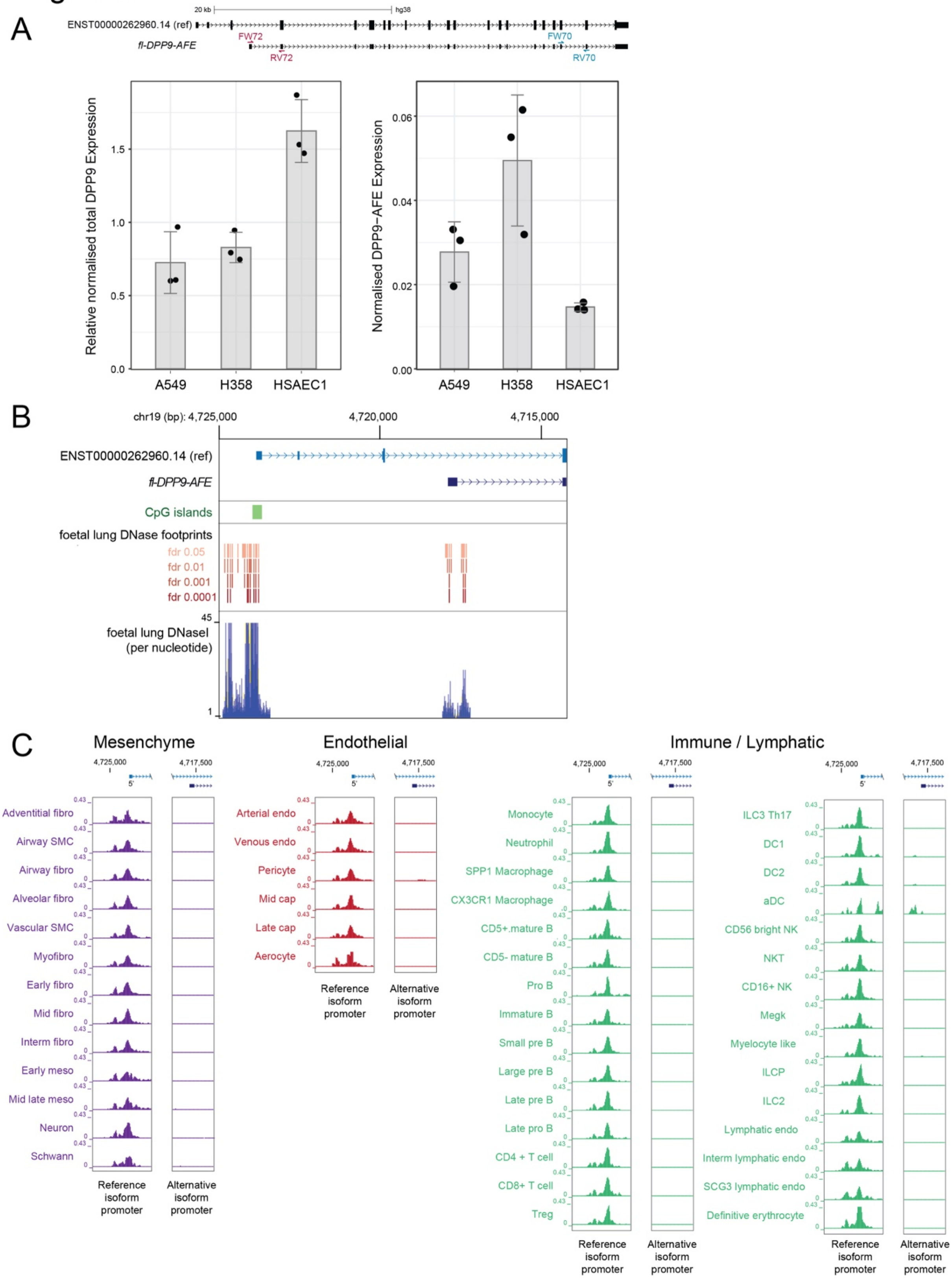

Figure S9. A DPP9 transcript isoform expression and regulation
A. Schematic showing primer annealing sites for forward (FW70) and reverse (RV70) primers used to amplify all *DPP9* transcripts (left panel) and for forward (FW72) and reverse (RV72) primers used to amplify the DPP9-AFE isoform (right panel). For all

DPP9 mRNA isoforms (left) or the *DPP9-AFE* mRNA isoform (right), expression was normalized to *GAPDH* and *SAFB* expression in A549, H358 and HSAEC1 lung epithelial cell lines. n = 3 biological replicates for each cell line.
B. Genome browser image of the reference transcript promoter, and *fl-DPP9-AFE* alternative promoter, showing location of CpG islands, and ENCODE DNaseI hypersensitive sites and footprints (at various false discovery rates; FDR), from foetal lung tissue [102].
C. Genome browser image of published single-cell ATAC-seq [59] tracks for mesenchyme, endothelial and immune/lymphatic cell types from foetal human lung. The pseudo-bulk ATAC-seq signal for the promoter of the reference transcript (left) and alternative promoter of the 19:4717660A>G variant-associated transcript isoform (right) are shown.
Source data are provided as a Source Data file.

Figure S10

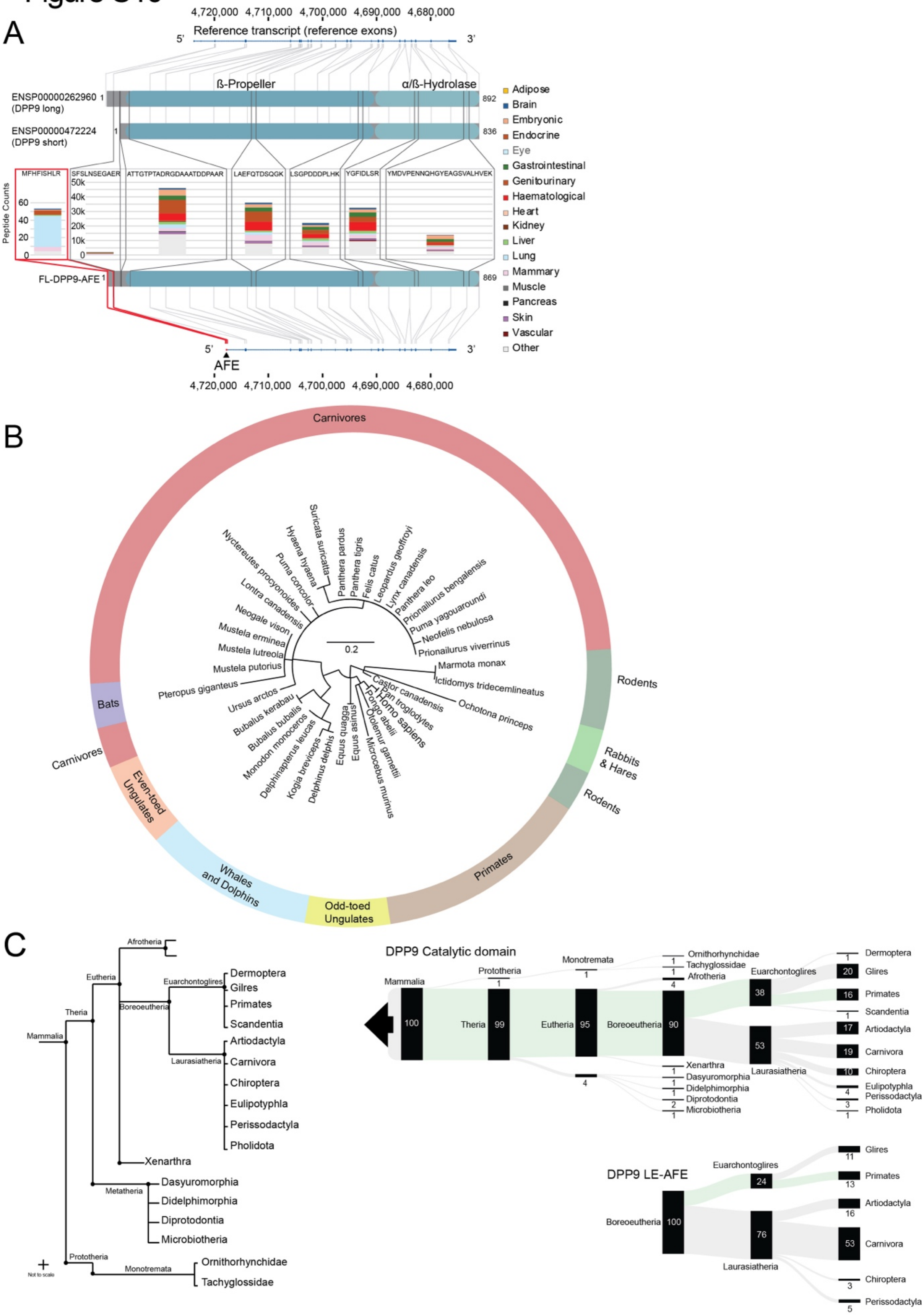

Figure S10. DPP9 protein expression and evolutionary conservation.

A. DPP9 peptide expression, including the AFE, and five highest detected peptides from proteomic meta-analysis [64] used to generate the background model.
B. Phylogenetic tree from maximum likelihood analysis of DPP9 alternative first exon peptide sequence matches from BLASTp.
C. Phylogenetic tree of mammalian clades associated with the DPP9 peptide phylogenetic ranges, and phylogenetic range of the DPP9 catalytic domain, with DPP9 alternate isoform alternative first exon encoding peptide. Clades determined from BLASTp using maximum likelihood analysis. The catalytic domain clades extend beyond Mammalia (Figure S11A). Green indicates the clades to *Homo sapiens*. Numbers represent percentages.
Source data are provided as a Source Data file.

Figure S11

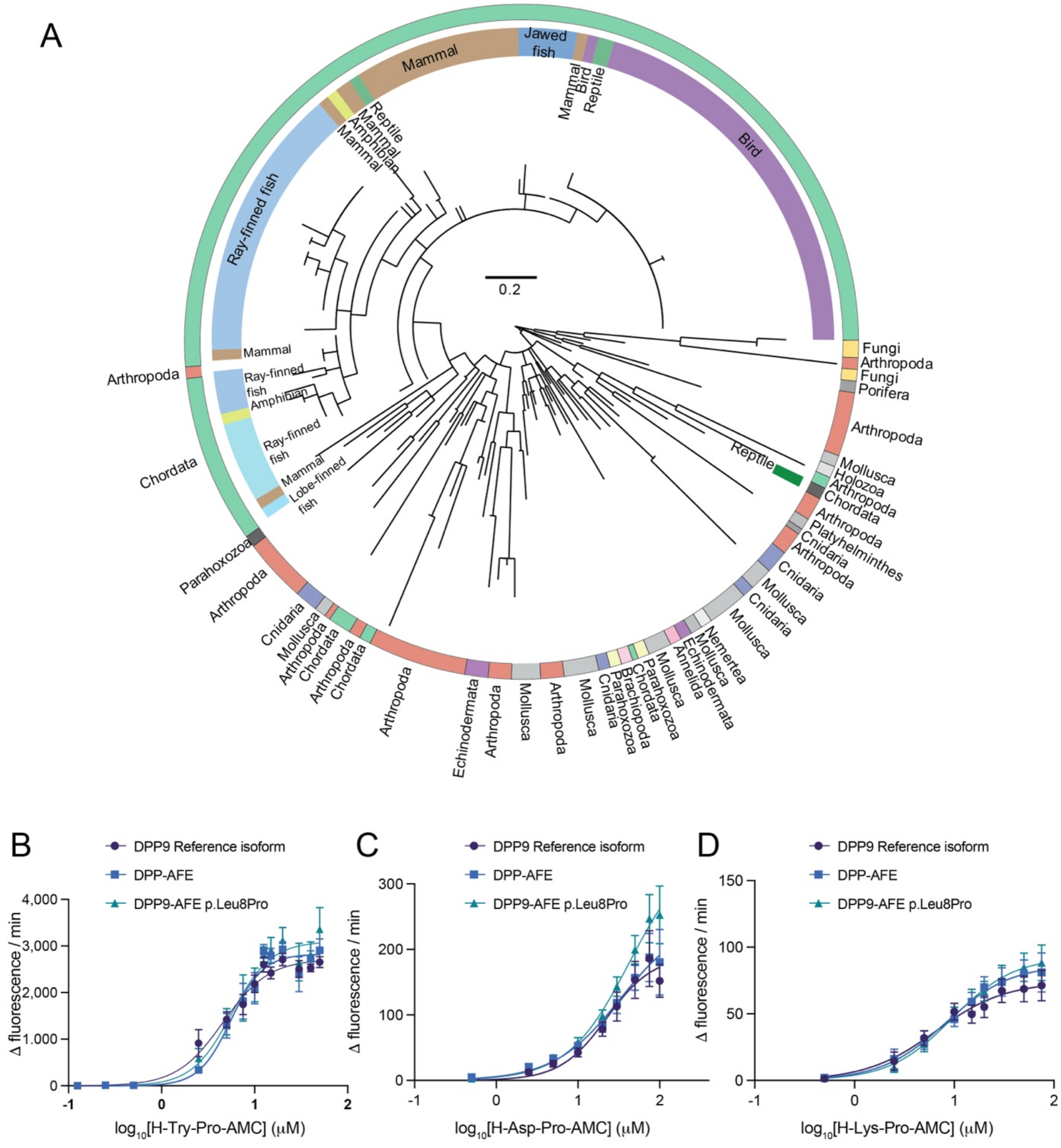

Figure S11. DPP9 isoform functional characterisation.
A. Phylogenetic tree from maximum likelihood analysis of DPP9 catalytic domain peptide sequence matches from BLASTp.
B, C, and D. DPP9-specific enzyme kinetics for DPP9 reference and AFE isoforms, and the p.Leu8Pro variant form of DPP9-AFE, using H-Try-Pro (B), H-Asp-Proline (C) and H-Lys-Pro-AMC (D) dipeptide substrates. Fluorescence emission was detected upon cleavage of the fluorogenic salt 7-amino-4-methylcoumarin (AMC), then the rate of hydrolysis data fit to an allosteric sigmoidal model. Mean with error bars showing ± SEM. Values were derived from three biological replicates (n = 3). See supplementary data 7 for details.
Source data are provided as a Source Data file.

Figure S12

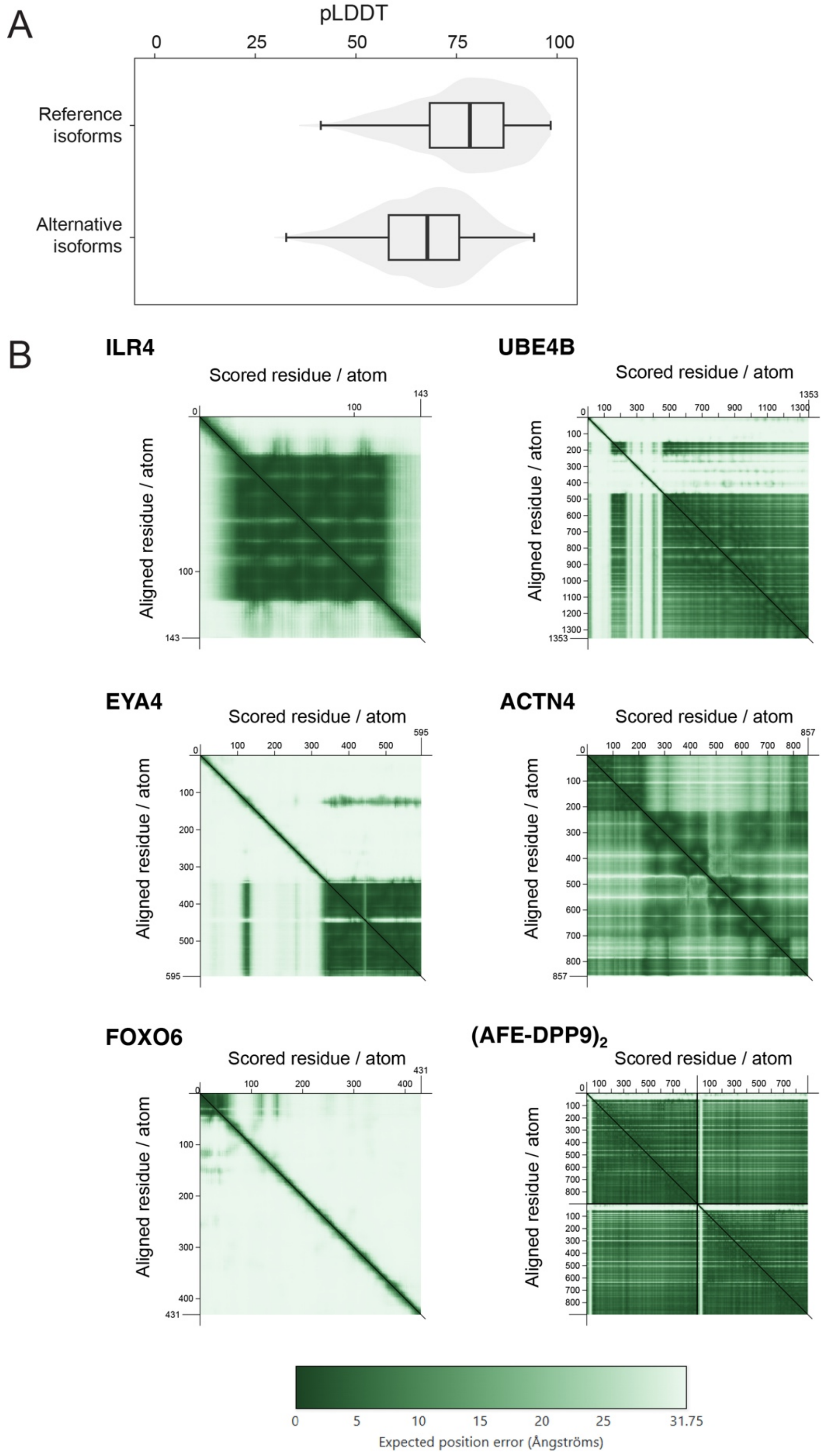

Figure S12. Alphafold3 per residue confidence and predicted aligned error scores.

A. Per residue confidence scores, as predicted Local Distance Difference Test (pLDDT) for reference (n = 2,484) and alternative isoform (n = 2,484) predicted structures.
B. Predicted aligned error (PAE) score for alternative isoforms depicted in figures 4E and S5.
Source data are provided as a Source Data file.

Supplementary Tables:

Table S1. Coding and Untranslated regions (UTR)

Table S2. Exon counts for ref-exons, and alt-exons.

Table S3. Alt-exons in catalogue (EIC) by alternative exon type: 3' splice site (3'SS), 5' splice site (5'SS), alternative first exon (AFE), alternative internal exon (including retained introns), and alternative last exon (ALE).

Table S4. Percent of repeat element types (LINE and SINE) for the exons per class that intersect repetitive sequences.

Table S5. Percent of Gencode (v46) annotated and unannotated isoforms for transcripts associated with variants from GWAS catalog and ClinVar in non-canonical exons in catalogue (EIC).

Table S6. Details for the examples of common and rare variants in alternative isoforms.

Table S7. Details for DPP9 enzymatic assays.

Table S8. Primer sequences used for RT-PCR.
