# Supplementary material for: Disease-associated genetic variants can cause missense effects in tissue-specific protein isoforms
Source: Nat Commun. 2026 Jun 16;17:7627. doi: 10.1038/s41467-026-74280-w (PMC13429693; doi:10.1038/s41467-026-74280-w)
Supplement: Supplementary file 2 — Description of Additional Supplementary Files [file 41467_2026_74280_MOESM2_ESM.pdf]

Description of Additional supplementary files

Supplementary Data 1: Exon counts for refexons, and alt-exons.

Supplementary Data 2: Alt-exons in catalogue (EIC) by alternative exon type: 3’ splice site (3’SS), 5’ splice site (5’SS), alternative first exon (AFE), alternative internal exon (including retained introns), and alternative last exon (ALE).

Supplementary Data 3: Coding and Untranslated regions (UTR)

Supplementary Data 4: Percent of repeat element types (LINE and SINE) for the exons per class that intersect repetitive sequences.

Supplementary Data 5: Percent of Gencode (v46) annotated and unannotated isoforms for transcripts associated with variants from GWAS catalog and ClinVar in non-canonical exons in catalogue (EIC).

Supplementary Data 6: Details for the examples of common and rare variants in alternative isoforms.

Supplementary Data 7: Details for DPP9 enzymatic assays.

Supplementary Data 8: Primer sequences used for RT-PCR.
