# Supplementary material for: Disease-associated genetic variants can cause missense effects in tissue-specific protein isoforms
Source: Nat Commun. 2026 Jun 16;17:7627. doi: 10.1038/s41467-026-74280-w (PMC13429693; doi:10.1038/s41467-026-74280-w)
Supplement: Supplementary file 4 — Reporting Summary [file 41467_2026_74280_MOESM4_ESM.pdf]

nature portfolio

Corresponding author(s): Simon C Biddie
Wendy A Bickmore
Joseph A Marsh

Last updated by author(s): May 14, 2026

Reporting Summary

Nature Portfolio wishes to improve the reproducibility of the work that we publish. This form provides structure for consistency and transparency in reporting. For further information on Nature Portfolio policies, see our Editorial Policies and the Editorial Policy Checklist.

Statistics

For all statistical analyses, confirm that the following items are present in the figure legend, table legend, main text, or Methods section.

n/a	Confirmed	
☐	☒	The exact sample size (n) for each experimental group/condition, given as a discrete number and unit of measurement
☐	☒	A statement on whether measurements were taken from distinct samples or whether the same sample was measured repeatedly
☐	☒	The statistical test(s) used AND whether they are one- or two-sided<br>*Only common tests should be described solely by name; describe more complex techniques in the Methods section.*
☒	☐	A description of all covariates tested
☐	☒	A description of any assumptions or corrections, such as tests of normality and adjustment for multiple comparisons
☐	☒	A full description of the statistical parameters including central tendency (e.g. means) or other basic estimates (e.g. regression coefficient) AND variation (e.g. standard deviation) or associated estimates of uncertainty (e.g. confidence intervals)
☐	☒	For null hypothesis testing, the test statistic (e.g. F, t, r) with confidence intervals, effect sizes, degrees of freedom and P value noted<br>*Give P values as exact values whenever suitable.*
☒	☐	For Bayesian analysis, information on the choice of priors and Markov chain Monte Carlo settings
☒	☐	For hierarchical and complex designs, identification of the appropriate level for tests and full reporting of outcomes
☐	☒	Estimates of effect sizes (e.g. Cohen's d, Pearson's r), indicating how they were calculated

Our web collection on statistics for biologists contains articles on many of the points above.

Software and code

Policy information about availability of computer code

Data collection	No software was used for data collection.

Data analysis	Ensembl VEP version 112<br>BLAST 2.<br>bigWigAverageOverBed v1.04<br>Gini coefficient using the ineq package R package v 0.2–13.<br>AlphaFold3 using a3m formatted alignments generated with MMseqs2 based on the UniRef and environmental sequence databases.<br>FoldX 5.0<br>GraphPad Prism 10.1.2<br>Bedtools v2.31.1<br>ONT MinKNOW 25.03.7, bream .8.4.4, configuration 6.4.10 and ONT MinKNOW Core 6.4.8, ONT Dorado v.7.8.3<br>IsoQuant v.3.7

For manuscripts utilizing custom algorithms or software that are central to the research but not yet described in published literature, software must be made available to editors and reviewers. We strongly encourage code deposition in a community repository (e.g. GitHub). See the Nature Portfolio guidelines for submitting code & software for further information.

Data

Policy information about availability of data

All manuscripts must include a data availability statement. This statement should provide the following information, where applicable:

- Accession codes, unique identifiers, or web links for publicly available datasets
- A description of any restrictions on data availability
- For clinical datasets or third party data, please ensure that the statement adheres to our policy

Data Generated: Genome expression Omnibus (GEO) repository under the accession number GSE303335
Data Used:
GEO Sequence Read Archive DataSets: A549 (SRR32141251, SRR32141252, SRR32141253); H358 (SRR24149739, SRR24149740, SRR24149741); HBEC3 (SRR32905582, SRR32905583, SRR32905584); HSAEC1-KT (SRR28773308, SRR28773309, SRR287733010); N/TERT-1 (SRR19142533, SRR19142536, SRR19142539)
Shi et al., 2024 (FLIbase) version 4.0 (http://www.flibase.org/#/download)
PhyloP-470way bigwig files were obtained from UCSC genome browser (v1.04.00)
RepeatMasker track from the UCSC genome browser for Hg38
gnomAD v4.1
dbSNP 155
ClinVar (VCF weekly release 2024-08-05)
Gencode V46

Data availability:

FLEXIR-Seq data has been deposited in the Genome expression Omnibus (GEO) repository under the accession number GSE303335 [https://www.ncbi.nlm.nih.gov/geo/query/acc.cgi?acc=GSE303335]. A web portal for gene or variant queries can be performed at https://genesis.igc.ed.ac.uk/. The exon class files, and variant annotations for ClinVar and GWAS variants mapping to alternative exons, with ESM-1v and Alphafold3-FoldX ΔΔG scores, are publicly available [https://github.com/sbiddie/Alternative_exons]. Alphafold3 structures for alternative isoforms have been deposited in the OSF repository [https://osf.io/btp73]. Source data are provided with this paper.

Research involving human participants, their data, or biological material

Policy information about studies with human participants or human data. See also policy information about sex, gender (identity/presentation), and sexual orientation and race, ethnicity and racism.

Reporting on sex and gender	Not applicable
Reporting on race, ethnicity, or other socially relevant groupings	Not applicable
Population characteristics	Not applicable
Recruitment	Not applicable
Ethics oversight	Not applicable

Note that full information on the approval of the study protocol must also be provided in the manuscript.

Field-specific reporting

Please select the one below that is the best fit for your research. If you are not sure, read the appropriate sections before making your selection.

☒ Life sciences ☐ Behavioural & social sciences ☐ Ecological, evolutionary & environmental sciences

For a reference copy of the document with all sections, see nature.com/documents/nr-reporting-summary-flat.pdf

Life sciences study design

All studies must disclose on these points even when the disclosure is negative.

Sample size	For experiments performed, sample sizes are as below:<br>For Full-Length targEted capture using eXon probes for IsofoRms (FLEXIR-seq), experiments from cell lines were performed in duplicate or triplicate.<br>For DPP9 enzymatic experiments, substrate experiments used three biological replicates, measured in technical duplicates or triplicates as indicated, while inhibitor experiments were performed as six biological replicates using technical duplicates.<br>For co-immunoprecipitation experiments, immunoblots were performed in triplicate. Representative blots and uncropped images are provided.<br>The sample numbers above are in keeping with the biological experiments required for the given assay. No sample size calculation was performed.<br><br>For computational analyses of existing data, the complete dataset was considered where applicable. For GTEx long-read RNA-seq data, the maximumnumber of replicates available per tissue were used. For GnomAD data, allelic frequencies were determined from the entire available population.
Data exclusions	For GnomAD data, only variants with exome and whole genome data were included.<br>For isoform analysis: only coding isoforms were considered, and excluded nonsense mediated decay trancripts where indicated, and for expression data only transcripts that are expressed in GTEx long-read RNA-seq data were included.<br>For variant analysis, spliceAI scores of > 0.2 were excluded to minimise variant effects secondary to splicing.
Replication	Experimental findings were replicated by biological replicates.<br>For Full-Length targEted capture using eXon probes for IsofoRms (FLEXIR-seq), experiments from cell lines were performed in duplicate or triplicate.<br>For DPP9 enzymatic experiments, substrate experiments used three biological replicates, measured in technical duplicates or triplicates as indicated, while inhibitor experiments were performed as six biological replicates using technical duplicates.<br>For co-immunoprecipitation experiments, immunoblots were performed in triplicate. Representative blots and uncropped images are provided.
Randomization	Not applicable. No randomization was performed for computational or experimental methods. For experiments using cell cultures, covariates were controlled using isogenic cell lines, with only the DPP9 isoform being the variable tested as experimental groups.
Blinding	No blinding for data collection or analysis was performed due to practical or methodological constraints of the computational and experimental analyses.

Reporting for specific materials, systems and methods

We require information from authors about some types of materials, experimental systems and methods used in many studies. Here, indicate whether each material, system or method listed is relevant to your study. If you are not sure if a list item applies to your research, read the appropriate section before selecting a response.

Materials & experimental systems

n/a	Involved in the study
☐	☒ Antibodies
☐	☒ Eukaryotic cell lines
☒	☐ Palaeontology and archaeology
☒	☐ Animals and other organisms
☒	☐ Clinical data
☒	☐ Dual use research of concern
☒	☐ Plants

Methods

n/a	Involved in the study
☒	☐ ChIP-seq
☒	☐ Flow cytometry
☒	☐ MRI-based neuroimaging

Antibodies

Antibodies used	α-GFP, Proteintech #PABG1, 1:1000 dilution - Polyclonal, (lot no: 70828032AB)<br>α-Flag, Sigma #F1804, 1:750 dilution - Clone M2, (lot no:. 000030821)<br>α-GAPDH, Proteintech 10494-1-AP, 1:1000 dilution - Polyclonal (lot no:N/A)<br>Anti-rabbit IgG, HRP-linked Antibody, Cell Signalling, 7974S (lot no: 29)<br>Anti mouse IgG, HRP linked Antibody, Cell Signalling, 7076S (lot no: 38)
Validation	As specified in https://www.abcam.com/en-us/products/primary-antibodies/dpp9-antibody-catalytic-domain-ab42080 and https://www.abcam.com/en-us/products/primary-antibodies/hrp-beta-actin-antibody-ac-15-loading-control-ab49900

Eukaryotic cell lines

Policy information about cell lines and Sex and Gender in Research

Cell line source(s)	A549 cells (ATCC, CCL-185)<br>HEK293T cells (ATCC, CRL-3216)<br>H358 cells (ATCC, CRL-5807)<br>HCT116 cells (ATCC, CCL-247)<br>HSAEC1-KT cells (ATCC, CRL-4050)<br>N/TERT-1 keratinocytes (gift from Jim Rheinwald)
Authentication	No cell lines were formally authenticated
Mycoplasma contamination	Cell line were tested negative for mycoplasma contamination.
Commonly misidentified lines<br>(See ICLAC register)	None

Plants

Seed stocks	Not applicable.
Novel plant genotypes	Not applicable.
Authentication	Not applicable.
