# Supplementary material for: Disease-associated genetic variants can cause missense effects in tissue-specific protein isoforms
Source: Nat Commun. 2026 Jun 16;17:7627. doi: 10.1038/s41467-026-74280-w (PMC13429693; doi:10.1038/s41467-026-74280-w)
Supplement: Supplementary file 5 — Transparent Peer Review file [file 41467_2026_74280_MOESM5_ESM.pdf]

nature portfolio

Peer Review File

Disease-associated genetic variants can cause missense effects in tissue-specific protein isoforms

Corresponding Author: Dr Simon Biddie

This file contains all reviewer reports in order by version, followed by all author rebuttals in order by version.

Version 0:

Reviewer comments:

Reviewer #1

(Remarks to the Author)
The manuscript presents an interesting exploration of tissue-specific isoforms and their potential contribution to variant pathogenicity; however, several aspects of the methodology, interpretation, and presentation require clarification and refinement. I do not believe the analyses as they stand demonstrate that the authors “framework can identify functional variants, and prioritize functional alternative isoforms, distinguishing these from the broader background of transcript noise” as they state in their discussion.
My major comments center around the choice and justification of statistical measures (e.g., the use of Gini coefficients for tissue specificity), the appropriateness of variant sets used in enrichment analyses, and the interpretation of pathogenicity evidence from ClinVar and GWAS variants. In addition, several figures lack appropriate statistical support or comparative context, making it difficult to evaluate the robustness of the claims. Finally, some methodological choices—such as the treatment of splicing consequences in alternative isoforms—require further justification to ensure that the conclusions are biologically meaningful rather than artifacts of the chosen approach. Alongside these points, I also provide a set of minor comments related to figure presentation, consistency, and clarity of reporting.

1. The authors use Gini coefficients to quantify tissue specificity. Why Gini? I had a quick glance at the paper they reference which benchmarks various methods and it seemed to recommend the use of the tau measure rather than gini. Can the authors justify/validate that the Gini coefficients are meaningful measures and not just driven by stochastic noise at low expression levels.

I think if an alternative transcript is expressed at a lower level than a reference it will necessarily be ‘more tissue specific’ but I’m not sure if this is really meaningful- what does it mean? If a transcript is expressed at TPM 5 in blood and TPM 1 in liver, and another transcript is expressed 10x lower in both tissues, it will drop below the level of detectability in liver, but is it any more meaningfully ‘tissue-specific’? It’s expressed at the same relative amount in different tissues. What are the authors really trying to measure and how does it relate to variant pathogenicity?
2. “We find that GWAS variants are enriched in alt-exons across biological systems”- in the legend for figure S2A it says the expected numbers were generated from ‘ten imputations of randomly selected genomic variants (expected)’. This is a somewhat meaningless comparison. The GWAS variants in the GWAS catalog are just the most significant hit at each loci- not necessarily a causal variant. The enrichments seen in figure S2A could easily (and likely do) just reflect the enrichment for variants near genes expressed in relevant tissues to the trait. Later in the paper the authors use finemapped credible sets for a different analysis- this would be a much better set of variants to try to assess enrichment on. I believe the ‘baseline’ expected number of variants should be based on the expectation that variants in the reference transcript are driving the signal, so that the authors can assess if the alternative transcript specifically has any evidence for biological/disease relevance.
3. Figure 2D needs some statistics- the authors say ‘ClinVar pathogenic variants are the most damaging according to both metrics’ however I don’t see that on the plot- pathogenic look very similar to benign, but it’s very hard to compare the groups on this plot especially with no statistics.
4. I’m also not convinced by the analysis of ClinVar pathogenic variants- what are these variants? with only 18, the authors could easily do a systematic check of how they were classified as pathogenic- I suspect many of them could be classified for their impact on splicing- i.e. pseudoexon creating intronic variants. In that case assessing them as missense variants on alternative transcripts is misleading.
5. The authors “highlight several cases in which disease-associated variants map to tissue-specific alternative isoforms and

are predicted to be damaging". In Figure S2E they plot the TPM of the alternative isoforms for 4 genes across tissues. However they do not plot the reference isoform- I think it's important to interpretation of the possible biological relevance/role of these alternative transcripts to be able to compare their expression. For instance the FOXO6 alternative isoform- is the reference isoform also expressed primarily in Ovary? Or a different tissue?
6. They go on in the same paragraph to list several GWAS hits which map to alternative transcripts- however this has the same methodological issues as in my second point. Are the variants they use fine-mapped hits with high PIP? Or just the variant with the lowest P-value at the locus? If the second, this analysis is quite meaningless.
7. The authors discuss VUS in an alternative exon of EYA4 which high sequence homology to a downstream ref-exon. In Figure S3D they show VEP scores for the VUS in the alternative isoform, however I feel this analysis would be much stronger if they had some meaningful comparison. For instance- are there clinically classified variants in the downstream ref-exon with sequence homology? If so, do the VUS look more similar to pathogenic or benign variants? What about for the rest of the gene? Fig. S3E shows the expression for the transcript across tissues, but again I was wondering if the expression across tissues is any different to the ref transcript- I looked quickly on GTEx and it looked quite similar but that was just at a glance.
8. I'm unclear on what the expected count of variants from Figure S4A is coming from – the legend says 'equal number of randomly selected genome variants to the observed variant in exon classes'. I'm not sure what this is referring to, and if it is a meaningful analysis.
9. I'm not sure what Figure 3A is intended to show – I think if the authors are trying to show MAF vs the variants likely causal they should be plotting PIPs rather than p-values. They say "Co-localization analysis suggests chr19:4717660-A>G is causative for COVID-19"- surely not?
10. In the methods when they discuss 'variant effect prediction in isoforms', they say they use masked SpliceAI scores to discard variants with a splice consequence in the ref-exon transcript. Do they consider variants with a splice consequence on the alt-transcript? Missense variants in the alt transcript could also be decreasing the strength of the splice-sites of the alternative exons. I don't think masked SpliceAI scores would show if they were as it would be a decrease in strength of an 'unannotated' site (relative to the ref transcript). The authors should discuss if they considered possible splicing consequences for the alt transcript as an alternative explanation for why they may be damaging.

Minor Comments
- The legend for Figure S2 is labelled with the wrong panels- E for F etc.
- The authors analyse a 'rare GWAS variant linked to eosinophil counts' however I couldn't find a reference for the GWAS they got it from
- For 'rare' GWAS hits the authors don't list the MAFs- how rare? This is quite a vague word. It would be better to list the MAFs, both in the text and in the supplementary table.
- Fig. S4A- I'm not sure a bar plot is appropriate as it's an obs/expected ratio- shouldn't the bars at least originate at 1 rather than 0?
- In the sentence 'The EIC variants in alt-exon have a range of MAFs, most of which (94/98) have a GWAS p-value of > 1x10-15' I assume the authors meant p-value of < 1x10^-15?

Reviewer #2

(Remarks to the Author)
We congratulate the authors on an important study that demonstrates the need to consider alternate, and sometimes tissue-specific, transcripts when interpreting potential disease-associated variants. Their examination of the properties of non-reference exons present in transcripts identified through prior long-read transcriptomic studies provides insight into the origin of some of the alternative and tissue-specific transcripts observed in humans. The identification of disease-associated variants that are likely to act through alternative or tissue-specific transcripts further highlights the need to look beyond the MANE-select transcript when interpreting variants.
The authors also describe a comprehensive framework for characterizing the expression of different gene transcripts using targeted long-read sequencing. They demonstrate the utility of this framework in studies of alternative transcripts in disease by showing that a GWAS variant associated with COVID-19 severity affects a coding region of a lung epithelial tissue-specific DPP9 transcript that is absent from the reference/MANE-select transcript. This in-depth analysis also builds on their other analysis of disease-associated variants in alternative transcripts, providing the strongest evidence in this work for a disease associated variant that acts through a tissue-specific transcript.
We believe the results of the manuscript to be of interest, but some analyses and results could be better presented and explained, to make the manuscript clearer to the readers.

Major Comments:
1. The exon conservation data presented by the authors is interesting but feels underutilised later in the paper. Currently, this section does not tie in well with the other analyses in the paper, as it includes exons not used in the other analyses (the not-in-catalogue exons) and is only briefly discussed without linking it to the other results. Some additional analyses could improve this. For example, it would be useful to see how the conservation scores differ in exons that show higher constraint or contain disease-associated variants or if alt-exons and ref-exons with similar conservation scores have similar properties (e.g. proportion of variant types in GnomAD, constraint, etc.).
2. For the AlphaFold3 models, it is unclear from the methods whether the per residue confidence scores or domain position confidence at either the variant site or for the entire protein were considered in the analysis and if any QC of the predicted structures took place. AlphaFold3 models vary significantly in their accuracy at the protein and domain level. Additionally, it would be important to know if the RepairPDB and BuildModel commands were also applied to the WT models as they may introduce differences to the Gibbs free energy when compared to the initial output of AlphaFold3, regardless of the amino

acid change. Finally, where the paper includes example AlphaFold3 models in the results, it would be helpful to include the predicted aligned error plots in the supplementary material.

Minor Comments:
3. Where existing datasets have been used, it is not always clear which analyses have been performed by the authors and which by the creators of the database. For example, for the isoform data from Shi et al., which is available through the FLIBase database, it is unclear if the authors performed additional QC on the transcripts from the database, or if they used the complete transcript list from the database
4. It is unclear why the authors decided to only include missense variants when examining the effect of disease associated variants on non-reference transcripts. Stop-gain and frameshift variants that occur in alternative transcripts are likely to have a more severe effect on the transcript's function and could knock out expression of the transcript entirely. Instances of disease associated variants that uniquely affect non-reference transcripts would be interesting and would not require the same degree of in-silico analyses to corroborate their deleteriousness as the discussed missense examples.
5. While the decision to exclude exons not in catalogue from the variants analysis is included in the limitations, the justification for doing so should be expanded upon. It is not clear why these exons were excluded from the analysis, given the earlier analysis shows they represent approximately half of all non-ref exons.
6. The phrasing of the title could be improved slightly for accuracy – the genetic variants don't really cause mutations as they are themselves the mutations.
7. To avoid confusion, it may be worth altering the abbreviation VEP, as it is not referring to the ensembl VEP software which has become synonymous with that abbreviation.
8. Alternate exons can frequently be non-coding, altering the 5' and 3' UTRs of a gene. It would be helpful to know the breakdown of coding and non-coding exons identified in the initial analysis and how they differ in conservation.
9. The authors should consider clarifying the difference between high-impact variants which cause monogenic disease (which could be classified as disease-causing variants) vs disease-associated GWAS variants which would be predicted to have a smaller impact, since their findings have different implications for these 2 groups of variants (missed diagnoses for the first, insights into GWAS biology in the second), both in the introduction/ discussion and in the presentation of the results. For example, the paragraph starting at line 229 highlights a series of disease-associated variants mapping to tissue-specific alternative isoforms. Whilst some are clearly GWAS variants, it is less clear if some of the other examples are disease-associated variants increasing the risk of a common phenotype or variants assessed within a clinical setting as possibly causing a monogenic disease (which would be most ClinVar variants).
10. Linked to the point above, odds ratios for disease-associated variants would be useful to give an indication of the predicted impact of the variants on disease risk
11. The authors may want to consider moving Panels E and F from figure 2 to the supplementary, the size of the panels make the variant position hard to interpret and the nature of the protein region that the variant is found in is clear from the text.
12. The $\Delta\Delta G$ values are somewhat difficult to interpret without a reference - it would be useful to have a range of values for known pathogenic missense variants in reference transcripts to compare it to.

Conclusions:
Overall, this work has an important message which would be relevant to the readership of Nature Communications. The evidence that some disease-associated variants act through non-reference and tissue-specific transcripts has important implications for variant interpretation and the FLEXIR-seq framework has potential utility in other studies of the role of tissue-specific transcripts in both rare and common diseases.

Reviewer #3

(Remarks to the Author)
I co-reviewed this manuscript with one of the reviewers who provided the listed reports. This is part of the Nature Communications initiative to facilitate training in peer review and to provide appropriate recognition for Early Career Researchers who co-review manuscripts.

Reviewer #4

(Remarks to the Author)
This is a very beautiful and comprehensive piece of work that brings to light an important missing piece in the interpretation of disease-associated variants. I think the authors did an excellent job at presenting a very complex study, and I only have some very minor comments on the manuscript. I also have some comments on the figures that I believe would improve the final version of the paper.

Comments on manuscript
I think that there are quite a few methodological details that are brought into the Results section, and perhaps could be removed to help with readability.

Could the authors comment on how much this paper is shifting the paradigm that most GWAS variants are non-coding?

line 120: are all the transcripts included here combined from public data?

line 135: What is the distribution of alternative exons only? How many per gene?

line 140: too much detail for a results section?

line 181: it would be interesting to compare this figure (~40K variants in alt-exons) to the number of variants found in ref-exons to give it more context

lines 185-187: is this sentence referring to the right figure? Why was this performed for respiratory and endocrine traits specifically?

lines 198-200: is this sentence referring to the right figure?

line 531: do the AF bins include any of the values? For example, does the “common” bin include the 0.05 or 0.5 values?

Figures
While I appreciate that there is a lot that needs to be summarised in this paper, I think there are too many panels for each figure, making them difficult to digest. Also there is a lot of back and forth between main and supplementary figures, so maybe the authors could reflect again on how to help the reader navigate this by perhaps moving some of them. I am making some more specific suggestions below.

Fig. 1:
the text is very small, difficult to read even when zoomed in
panels B and D could be tables, leaving space for the rest of the figure

Fig. 2:
panel C can be a table

Fig. 3
I would suggest moving panel C to supplementary materials

Fig. 4
panel A can be a table
panel D is not cited in the main text

Fig. S1
panels A and E could be tables

Fig. S2
panel E in completely unreadable and panel A is not much better

Fig. S3
panel D can be a table
panel E is difficult to read

Fig S4
panel A can be just described in the text
panel E would it be worth commenting on the correlation between different cell lines?

Version 1:

Reviewer comments:

Reviewer #1

(Remarks to the Author)
I thank the authors for their thorough and gracious response to my comments. The revised manuscript is substantially improved, and the authors have addressed all of my major and minor concerns & reservations about the analyses. I am satisfied with the revisions and recommend acceptance of the manuscript. Congratulations to the authors on a very interesting study that i'm sure will be of broad interest and utility to the genetics community!

One extremely tiny typographic correction: in the discussion the DPP9 variant is referred to as "chr19:1717660-A>G" rather than "chr19:4717660-A>G", which is used consistently throughout the rest of the manuscript. This should be corrected prior to publication.

Reviewer #2

(Remarks to the Author)
We are happy with the changes that the authors made in response to our comments. We believe the manuscript is now more clear and do not have any further comments. We would like to congratulate the authors on their work.

Reviewer #3

(Remarks to the Author)
I co-reviewed this manuscript with one of the reviewers who provided the listed reports. This is part of the Nature Communications initiative to facilitate training in peer review and to provide appropriate recognition for Early Career Researchers who co-review manuscripts.

Reviewer #4

(Remarks to the Author)
I think the paper has improved in readability thanks to all the changes implemented following the reviewers' comments. My concerns have been addressed and I am satisfied with the modifications and the answers provided.

Open Access This Peer Review File is licensed under a Creative Commons Attribution 4.0 International License, which permits use, sharing, adaptation, distribution and reproduction in any medium or format, as long as you give appropriate credit to the original author(s) and the source, provide a link to the Creative Commons license, and indicate if changes were made.
In cases where reviewers are anonymous, credit should be given to 'Anonymous Referee' and the source.
The images or other third party material in this Peer Review File are included in the article’s Creative Commons license, unless indicated otherwise in a credit line to the material. If material is not included in the article’s Creative Commons license and your intended use is not permitted by statutory regulation or exceeds the permitted use, you will need to obtain permission directly from the copyright holder.
To view a copy of this license, visit https://creativecommons.org/licenses/by/4.0/

Nature Communications NCOMMS-25-70581-T

Response to reviewers:

We thank the reviewers for their comments, which we believe have helped us significantly improve the manuscript. We have addressed all reviewer comments below, resulting in changes to figure layout, changes to text to provide clarification, and new analyses. We have increased the number of main figures, supplementary figures, and supplementary tables.

In addition, we have performed experiments, providing evidence for functional differences of the DPP9-AFE isoform compared to the reference isoform. We observe the DPP9-AFE isoform to have a higher affinity for the DPP inhibitor, Val-boroPro, with the common DPP9 variant increasing this affinity further. This agrees with our previous finding of differences in enzymatic activity with the structurally similar H-Val-Pro dipeptide. The new data are shown in the new Figure 5.

REVIEWER COMMENTS

Reviewer #1:

The manuscript presents an interesting exploration of tissue-specific isoforms and their potential contribution to variant pathogenicity; however, several aspects of the methodology, interpretation, and presentation require clarification and refinement. I do not believe the analyses as they stand demonstrate that the authors “framework can identify functional variants, and prioritize functional alternative isoforms, distinguishing these from the broader background of transcript noise” as they state in their discussion.
My major comments center around the choice and justification of statistical measures (e.g., the use of Gini coefficients for tissue specificity), the appropriateness of variant sets used in enrichment analyses, and the interpretation of pathogenicity evidence from ClinVar and GWAS variants. In addition, several figures lack appropriate statistical support or comparative context, making it difficult to evaluate the robustness of the claims. Finally, some methodological choices—such as the treatment of splicing consequences in alternative isoforms—require further justification to ensure that the conclusions are biologically meaningful rather than artifacts of the chosen approach. Alongside these points, I also provide a set of minor comments related to figure presentation, consistency, and clarity of reporting.

1. The authors use Gini coefficients to quantify tissue specificity. Why Gini? I had a quick glance at the paper they reference which benchmarks various methods and it seemed to recommend the use of the tau measure rather than gini. Can the authors justify/validate that the Gini coefficients are meaningful measures and not just driven by stochastic noise at low expression levels.

We have updated the manuscript with 3 newer references to support the use of Gini coefficients. These report that: 1) Gini coefficients reflect underlying tissue-specific gene expression distribution compared to the Tau value (Vorperian et al., 2022; doi.org/10.1038/s41587-021-01188-9), 2) Gini coefficients robustly identify ‘house-keeping’ genes by measuring uniform expression using large datasets from cell-lines and tissues (Muelas et al., 2019, doi.org/10.1038/s41598-019-54288-7), and 3) the use of Gini coefficient in GTEx long-read RNA-sequencing (Page et al., 2024, doi.org/10.1101/2024.02.13.579945).

I think if an alternative transcript is expressed at a lower level than a reference it will necessarily be 'more tissue specific' but I'm not sure if this is really meaningful- what does it mean? If a transcript is expressed at TPM 5 in blood and TPM 1 in liver, and another transcript is expressed 10x lower in both tissues, it will drop below the level of detectability in liver, but is it any more meaningfully 'tissue-specific'? It's expressed at the same relative amount in different tissues. What are the authors really trying to measure and how does it relate to variant pathogenicity?

The reviewer raises an important point. The Gini coefficient does not account for the expression level of the transcript being measured relative to other transcripts of the same gene, such as the reference isoforms. We find that both the gini coefficient and expression (TPM) is higher in the alternative transcript isoform compared to the reference in 20% of cases. This proportion is similar when considering transcripts that are associated with missense variants from GWAS and ClinVar. Therefore, a proportion of alternative transcripts have both high expression relative to the reference isoform and higher tissue-specificity. In this case, coding variants in alternative isoforms that are associated with high expression (close or similar to the reference isoform) may contribute to disease.

In view of this, we have added on line 198 "Together, 20% of alternative transcripts had higher tissue-specificity and higher expression than the reference isoform." We have provided an additional plot, in figure S2C, to show the alterative to reference ratios for gini coefficient and expression (TPM). Additionally, in the discussion (line 435), we have added "Expression in the relevant tissue, and relative to the reference isoform. should be taken into account when considering the contribution of alternative isoforms to pathomechanisms."

2. "We find that GWAS variants are enriched in alt-exons across biological systems"- in the legend for figure S2A it says the expected numbers were generated from 'ten imputations of randomly selected genomic variants (expected)'. This is a somewhat meaningless comparison. The GWAS variants in the GWAS catalog are just the most significant hit at each loci- not necessarily a causal variant. The enrichments seen in figure S2A could easily (and likely do) just reflect the enrichment for variants near genes expressed in relevant tissues to the trait. Later in the paper the authors use finemapped credible sets for a different analysis- this would be a much better set of variants to try to assess enrichment on.

The enrichment analysis is based on a published method (Biddie et al., 2024, https://doi.org/10.1186/s13059-024-03352-1). The distribution of variants, eg. subsetted from dbSNP, are used as the expected distribution, with enrichment relative to genomic size of the feature. This has been used for functional regions including non-coding, and coding regions - in keeping with previous findings for enrichment of disease variants in coding regions (Gusev et al., 2014, https://doi.org/10.1016/j.ajhg.2014.10.004).

We agree with the reviewer that using fine-mapped variants would be a better approach, as we have done for COVID-19 variants, and emphasise this in the discussion. We now provide additional analysis using publicly available fine-mapped data from 14 Traits using the All of Us cohort (Rossen et al., 2026), and 94 traits using the UK BioBank cohort (Kiyose et al., 2021). In line with Reviewer 3 comment 10, about effect size, we provide plots of the PIP against beta for each cohort. We have also computed the enrichment in reference or alternative exons for the fine-mapped variants and find enrichment above expected for both ref-exons and alt-exons, but that there was no difference between the two classes. We have added these as figure S3C, and discussed the findings in the results section, line 207. We also caveat the GWAS catalog variants in the discussion as it is based on significance.

I believe the 'baseline' expected number of variants should be based on the expectation that variants in the reference transcript are driving the signal, so that the authors can assess if the alternative transcript specifically has any evidence for biological/disease relevance.

Our additional analysis of fine-mapped variants from All of Us and UKBB shows enrichment (approx. 2-fold) in both ref-exons and alt-exons, as discussed above. The enrichment analysis accounts for number of variants observed in each category - intended to show enrichment relative to the genomic size of the feature (ref- or alt-exons). For the aggregate fine-mapped variants from 14 traits (All of Us), or 94 traits (UKBB), we observed no difference in enrichment, but do see higher enrichment for alt-exons for COVID-19. Our fine-mapped variant plots for the PIP against beta would suggest that the assumption that the reference transcript is driving the signal is not consistently expected, with some alt-exon variants showing high PIP and beta values.

3. Figure 2D needs some statistics- the authors say ‘ClinVar pathogenic variants are the most damaging according to both metrics’ however I don’t see that on the plot- pathogenic look very similar to benign, but it’s very hard to compare the groups on this plot especially with no statistics.

We have added p-values to the figure - now Fig 2C, and the legend now reads “p-values were calculated using Pairwise Wilcoxon Rank Sum Tests with Benjamini-Hochberg (BH) correction”. The manuscript text has been updated (line 241) to state “As expected, ClinVar pathogenic variants are the most damaging, with ΔΔG having better discrimination”.

4. I’m also not convinced by the analysis of ClinVar pathogenic variants- what are these variants? with only 18, the authors could easily do a systematic check of how they were classified as pathogenic- I suspect many of them could be classified for their impact on splicing- i.e. pseudoexon creating intronic variants. In that case assessing them as missense variants on alternative transcripts is misleading.

For the 18 pathogenic variants identified (fig. 2C), we performed a manual review of the associated ClinVar entries and supporting literature to examine the basis for their pathogenic classification. We observe that 3/18 variants have evidence suggesting a possible splicing-related mechanism. For the other 15, the ClinVar annotations describe protein-level consequences in alternative isoforms and do not provide clear evidence for splicing effects in the reference transcript. We were able to obtain SpliceAI scores for 16/18 variants and observed a median score of 0.01 (range 0–0.19), which also suggests most are not likely to influence splicing, consistent with generally low predicted splice-altering potential under our filtering threshold. However, we agree with the reviewer that caution is required when interpreting exonic variants, as the primary functional consequence may in some cases occur through altered splicing rather than a protein-level change. To illustrate the structural context of variants in alternative isoforms, we focused on a subset of genes where the isoform structure and expression patterns allowed clear visualisation and interpretation (fig S4B); the full set of variants was nevertheless included in the quantitative analysis. We have added text in the results section to address this point and expanded on it in the discussion as a limitation of the study (lines 250, 513).

5. The authors “highlight several cases in which disease-associated variants map to tissue-specific alternative isoforms and are predicted to be damaging”. In Figure S2E they plot the TPM of the alternative isoforms for 4 genes across tissues. However they do not plot the reference isoform- I think it’s important to interpretation of the possible biological relevance/role of these alternative transcripts to be able to compare their expression. For instance the FOXO6 alternative isoform- is the reference isoform also expressed primarily in Ovary? Or a different tissue?

We have provided for comparison the reference transcript for FOXO6, ACTN4, IL4R and UBE4B, and EYA4. Following re-arrangement of figures as suggested by reviewers, these now appear in figures S5 and S6. We agree that a comparison with the reference transcript

is important. For FOXO6, ACTN4, IL4R and UBE4B, the alternative isoform has higher expression in at least one tissue. For EYA4, the reference transcript has higher expression, but as we note in the discussion, the expression in disease states is not known. We have added text in the relevant results section to highlight the reference and alternative expression differences (lines 257).

6. They go on in the same paragraph to list several GWAS hits which map to alternative transcripts- however this has the same methodological issues as in my second point. Are the variants they use fine-mapped hits with high PIP? Or just the variant with the lowest P-value at the locus? If the second, this analysis is quite meaningless.

We have provided a new figure 3A, which plots the MAF vs PIP and includes the GWAS p-values in colour-coding. The DPP9 variant, which was taken forward, has a high PIP and GWAS p-value for the locus. We have modified the text with the additional PIP information. We have provided Alt-exon annotated fine-mapped variants for complex traits from UKBB and All of Us in figure S3C. With the information provided by the GWAS catalog, we cannot state the PIP, but our approach provides a method for others to consider alternative exons as a signal for complex traits.

7. The authors discuss VUS in an alternative exon of EYA4 which high sequence homology to a downstream ref-exon. In Figure S3D they show VEP scores for the VUS in the alternative isoform, however I feel this analysis would be much stronger if they had some meaningful comparison. For instance- are there clinically classified variants in the downstream ref-exon with sequence homology? If so, do the VUS look more similar to pathogenic or benign variants? What about for the rest of the gene?

The downstream exon which is retained in the reference isoform does not have known pathogenic variants to compare. As such, we have updated the figure (now S6D) to show gnomAD variants that map to the alternative isoform, with both ESM-1v and $\Delta\Delta G$, as a comparator to the VUS found in the mutually exclusive exon. The VUS scores show more damaging effects than the gnomAD median for both ESM-1v and $\Delta\Delta G$ scores.

Fig. S3E shows the expression for the transcript across tissues, but again I was wondering if the expression across tissues is any different to the ref transcript- I looked quickly on GTEx and it looked quite similar but that was just at a glance.

We have provided new figures S5 and S6 to show the expression of the reference transcript with the alternative isoform across tissues. Please note that the long-read RNA-seq data on the GTEx web portal only displays annotated transcript isoforms, for both the splice junction and isoform expression views. This is evident, as an example, for DPP9, where the novel transcript we describe is not shown in the long-read RNA-seq GTEx web portal.

8. I'm unclear on what the expected count of variants from Figure S4A is coming from – the legend says 'equal number of randomly selected genome variants to the observed variant in exon classes'. I'm not sure what this is referring to, and if it is a meaningful analysis.

This figure is now described in the text as suggested by Reviewer #4. We have clarified the text to state that the enrichment is based on the expected distribution of variants relative to the genomic size of the feature (exon class) (line 291). This analysis is in line with comments from Reviewer #1 point 2, and based on a method we previously published to compute the enrichment of disease-variants in specific features - such as open chromatin, or exons - which have variable genomic size (Biddie et al., 2024, https://doi.org/10.1186/s13059-024-03352-1).

9. I'm not sure what Figure 3A is intended to show – I think if the authors are trying to show MAF vs the variants likely causal they should be plotting PIPs rather than p-values. They say "Co-localization analysis suggests chr19:4717660-A>G is causative for COVID-19"- surely not?

We have provided a new figure 3A, which plots the MAF vs PIP and includes the GWAS p-values in colour-coding. The DPP9 variant has a high PIP, and GWAS p-value for the locus. We have modified the text with the additional PIP information. We have also toned down the causative link from co-localization analysis, now stating that the variant "is shared in" COVID-19 and lung fibrosis.

10. In the methods when they discuss 'variant effect prediction in isoforms', they say they use masked SpliceAI scores to discard variants with a splice consequence in the ref-exon transcript. Do they consider variants with a splice consequence on the alt-transcript? Missense variants in the alt transcript could also be decreasing the strength of the splice-sites of the alternative exons. I don't think masked SpliceAI scores would show if they were as it would be a decrease in strength of an 'unannotated' site (relative to the ref transcript). The authors should discuss if they considered possible splicing consequences for the alt transcript as an alternative explanation for why they may be damaging.

In line with the above comment (number 4), we agree that exonic variants may operate through a splice altering mechanism, and cryptic or cell-type-specific splicing may be poorly predicted by current computational methods. Our analysis adopted a reference isoform-centric prioritisation strategy, with the aim of minimising the likelihood that a variant primarily affects the reference transcript. We sought to focus on missense variants, as the predominant class of coding variants, and the most challenging to interpret. To reduce the risk of misinterpreting variants that disrupt splicing of the reference transcript as missense effects in alternative isoforms, we applied a masked SpliceAI cutoff of 0.2 (chosen for high recall for splice-altering variants). This approach does not exclude the possibility that some variants may influence splicing of the alternative transcript itself, particularly where splice sites are not annotated in the reference transcript. Such cases cannot readily be filtered using masked SpliceAI scores. We mention this possibility in the results section related to ClinVar variants (line 248) and discuss it further as a limitation of the study (line 513). Importantly, whether the functional consequence occurs through altered splicing (e.g. causing exon inclusion) or through a protein-level change, the presence of the variant still implicates the alternative isoform as potentially relevant to the phenotype.

Minor Comments
- The legend for Figure S2 is labelled with the wrong panels- E for F etc.

The figures and figure legends have now been changed following reviewer comments.

- The authors analyse a 'rare GWAS variant linked to eosinophil counts' however I couldn't find a reference for the GWAS they got it from

We have added the relevant reference as annotated in the GWAS catalog (UK Biobank Whole-Genome Sequencing Consortium. Whole-genome sequencing of 490,640 UK Biobank participants. Nature 645, 692–701 (2025).

- For 'rare' GWAS hits the authors don't list the MAFs- how rare? This is quite a vague word. It would be better to list the MAFs, both in the text and in the supplementary table.

We have provided the MAF for the rare IL4R GWAS variant based on GnomAD MAFs. The MAF is 1.23x10^-6 for this variant (line 262). This has been amended in the supplementary table S6, where we also update MAFs for rare ClinVar variants based on GnomAD v4.1.0.

- Fig. S4A- I'm not sure a bar plot is appropriate as it's an obs/expected ratio- shouldn't the bars at least originate at 1 rather than 0?

We thank them for pointing this out. The figure has been changed, and is now S2D. Accordingly, we have also modified figure 4A.

- In the sentence 'The EIC variants in alt-exon have a range of MAFs, most of which (94/98) have a GWAS p-value of > 1x10-15' I assume the authors meant p-value of < 1x10^-15?

This has been corrected. In line with other comments, we have also added PIP values.

Reviewer #2 (Remarks to the Author):

We congratulate the authors on an important study that demonstrates the need to consider alternate, and sometimes tissue-specific, transcripts when interpreting potential disease-associated variants. Their examination of the properties of non-reference exons present in transcripts identified through prior long-read transcriptomic studies provides insight into the origin of some of the alternative and tissue-specific transcripts observed in humans. The identification of disease-associated variants that are likely to act through alternative or tissue-specific transcripts further highlights the need to look beyond the MANE-select transcript when interpreting variants.
The authors also describe a comprehensive framework for characterizing the expression of different gene transcripts using targeted long-read sequencing. They demonstrate the utility of this framework in studies of alternative transcripts in disease by showing that a GWAS variant associated with COVID-19 severity affects a coding region of a lung epithelial tissue-specific DPP9 transcript that is absent from the reference/MANE-select transcript. This in-depth analysis also builds on their other analysis of disease-associated variants in alternative transcripts, providing the strongest evidence in this work for a disease associated variant that acts through a tissue-specific transcript.
We believe the results of the manuscript to be of interest, but some analyses and results could be better presented and explained, to make the manuscript clearer to the readers.

Major Comments:
1. The exon conservation data presented by the authors is interesting but feels underutilised later in the paper. Currently, this section does not tie in well with the other analyses in the paper, as it includes exons not used in the other analyses (the not-in-catalogue exons) and is only briefly discussed without linking it to the other results. Some additional analyses could improve this. For example, it would be useful to see how the conservation scores differ in exons that show higher constraint or contain disease-associated variants or if alt-exons and ref-exons with similar conservation scores have similar properties (e.g. proportion of variant types in GnomAD, constraint, etc.).

The reviewer raises an important point, as conservation is frequently used to infer function, and have expanded analysis on the exons not-in-catalogue. We have addressed this in three ways. Firstly, we provide PhyloP-470way conservation scores for GWAS catalog variants, grouped by their mapping to ref-exons, EIC or ENIC alt-exons, 5'UTR, 3'UTR or non-coding regions (figure S2B). As expected, variants in ref-exons had the highest conservation, with variants in alt-exons and non-coding showing the lowest conservation. We have added these findings to the results section (line 194). Secondly, we plot conservation scores for GnomAD variants that map to exon classes, GWAS catalog variants, and ClinVar pathogenic, VUS and benign variants. GnomAD variants in alt-exons have similar conservation to GWAS variants. Meanwhile, pathogenic ClinVar variants have higher conservation compared to VUS and benign variants. This is shown in figure S3D and discussed in lines 217. Thirdly,

we have included conservation data on UTRs, (see point 8 below by this reviewer for further details). For UTRs, while conservation scores for EIC and ENIC alternative-exons are lower than ref-exons, they are >0, suggesting some evolutionary constraint (see figure S1C).

With respect to proportions, we mapped all GnomAD variants to Exons not-in-catalogue from long-read RNA-seq and find that synonymous effects dominate (81% of variants), followed by missense effects (15%), showing a higher percentage of synonymous effects compared to Ref-exon or EIC Alt-exons. We have updated figure 1D to include this.

2. For the AlphaFold3 models, it is unclear from the methods whether the per residue confidence scores or domain position confidence at either the variant site or for the entire protein were considered in the analysis and if any QC of the predicted structures took place. AlphaFold3 models vary significantly in their accuracy at the protein and domain level. Additionally, it would be important to know if the RepairPDB and BuildModel commands were also applied to the WT models as they may introduce differences to the Gibbs free energy when compared to the initial output of AlphaFold3, regardless of the amino acid change. Finally, where the paper includes example AlphaFold3 models in the results, it would be helpful to include the predicted aligned error plots in the supplementary material.

For the AF3 models of alternative isoforms, we analysed the predicted local distance difference test (pLDDT) metric, the commonly used confidence measure. We expected the alternative isoforms to have lower mean pLDDT than reference isoforms (figure S12A). We did not use pLDDT for filtering due to a number of caveats. Alternative isoforms often contain underrepresented sequences, and because pLDDT correlates with the depth and evolutionary information in the input multiple sequence alignment, this may introduce a bias towards sequences more similar to the reference isoform. However, we prioritised variants based on ΔΔG estimates, which are likely to represent damaging candidates since FoldX does not reliably evaluate disordered regions, which would typically correspond to low-confidence structural predictions. We augmented our analysis using ESM-1v predictions which are not dependent on structural confidence scores, to provide an additional layer of confidence in variant effect scoring. For the alternative isoforms presented, we have included predicted aligned error (PAE) plots (figure S12B). We have referenced this figure in the methods, to improve the flow of the manuscript as suggested by Reviewer 3.

With respect to the wild-type AF3 models, we used the method recommended by FoldX, where RepairPDB is called on the structure before predicting the ΔΔG of a mutation using the BuildModel command. We have clarified this procedure in the methods.

Minor Comments:
3. Where existing datasets have been used, it is not always clear which analyses have been performed by the authors and which by the creators of the database. For example, for the isoform data from Shi et al., which is available through the FLIBase database, it is unclear if the authors performed additional QC on the transcripts from the database, or if they used the complete transcript list from the database.

We have added clarification in the Defining exons classes section of the methods. We state in line 536 that the complete transcript models from the FLIbase database were used, and filtered for predicted amino acid sequences, nonsense mediated decay, and GTEx expression, where indicated in the text.

4. It is unclear why the authors decided to only include missense variants when examining the effect of disease associated variants on non-reference transcripts. Stop-gain and frameshift variants that occur in alternative transcripts are likely to have a more severe effect on the transcript's function and could knock out expression of the transcript entirely. Instances of disease associated variants that uniquely affect non-reference transcripts would

be interesting and would not require the same degree of in-silico analyses to corroborate their deleteriousness as the discussed missense examples.

To explain our focus, we have added a statement in the introduction to explain why we have focused on missense variants (line 104). Firstly, SNPs are the most prevalent type of genomic variation in coding regions (and indeed across the genome), and when they affect protein coding, they are more likely to be missense. Missense variants are the largest class for both pathogenic variants, and VUS. They are more challenging to interpret than severe outcomes like frameshift mutations etc., and as such have led to the development of variant effect models such as those utilised in our manuscript. By exploring alt-exons in-catalogue (EIC), variants that map to these are captured when using isoform-aware ensembl VEP, to identify more severe effects such as stop-gain, frameshift etc. This is shown in figure S3E for ClinVar variants.

5. While the decision to exclude exons not in catalogue from the variants analysis is included in the limitations, the justification for doing so should be expanded upon. It is not clear why these exons were excluded from the analysis, given the earlier analysis shows they represent approximately half of all non-ref exons.

When considering alternative exons, we have predominantly focused on exons in catalogue (EIC), excluding the exons not in catalogue (ENIC). We showed in fig S1C (now S1B) that ENICs had the lowest PhyloP conservation score, suggesting they are less likely to be functional. This observation agrees with a recent publication which identified "novel" exons from genes expressed in liver, measuring conservation using PhyloP, to show these novel exons had reduced conservation (Kiyose et al., 2022; 10.1371/journal.pgen.1010342).

Additionally, ENICs are not currently annotated in gene models e.g. Encode, do not have these "novel" exons, suggesting their expression may be lower. We provide an analysis (fig S2A) which shows that ENIC-containing transcripts have predominantly lower expression relative to Ref-exons and EIC, and a higher percentage are predicted to undergo nonsense mediated decay (NMD). Together, the lower conservation, low expression and higher NMD proportion, suggest ENIC transcripts are less likely to be functional and therefore variants are less likely to have major effects.

However, as shown in Kiyose et al., 2022 (10.1371/journal.pgen.1010342), some novel exons have high conservation, and are associated with expressed transcripts. Some ENICs may therefore have some role in diseases and genetic variation, which warrants further investigation in the future, but is beyond the scope of this manuscript.

We have added more details to explain the decision to exclude ENIC when considering disease-variant effects (line 190), and in the discussion as part of the limitations (line 514).

6. The phrasing of the title could be improved slightly for accuracy – the genetic variants don't really cause mutations as they are themselves the mutations.

We have modified the title to read "Disease-associated genetic variants can cause missense effects in tissue-specific protein isoforms"*.*

7. To avoid confusion, it may be worth altering the abbreviation VEP, as it is not referring to the ensembl VEP software which has become synonymous with that abbreviation.

We have changed all references to 'VEP' to now read 'variant effect scores'. All references specifically to ensembl VEP remain to avoid confusion.

8. Alternate exons can frequently be non-coding, altering the 5’ and 3’ UTRs of a gene. It would be helpful to know the breakdown of coding and non-coding exons identified in the initial analysis and how they differ in conservation.

The reviewer highlights an important point. While we have focused on coding effects of variants, UTR effects are known. For alternative isoforms, exons can indeed be non-coding UTRs, but this is transcript dependent, determined by the start codon position. We provide analysis of UTRs in table S3, showing that the fraction of exons that encode 5’and 3’ UTRs are much lower for alternative exons, than reference exons. A large fraction of reference and EIC alternative exons can be coding or are UTRs, depending on the transcript. We analysed conservation using PhyloP scores (figure S1C), for 5’ and 3’ UTRs in the exon classes (Ref-exons, EIC Alt-exon and ENIC Alt-exons), showing 5’UTRs of ref-exons have the highest conservation. The UTR analysis has been discussed in the first section of the results (line 137).

9. The authors should consider clarifying the difference between high-impact variants which cause monogenic disease (which could be classified as disease-causing variants) vs disease-associated GWAS variants which would be predicted to have a smaller impact, since their findings have different implications for these 2 groups of variants (missed diagnoses for the first, insights into GWAS biology in the second), both in the introduction/ discussion and in the presentation of the results. For example, the paragraph starting at line 229 highlights a series of disease-associated variants mapping to tissue-specific alternative isoforms. Whilst some are clearly GWAS variants, it is less clear if some of the other examples are disease-associated variants increasing the risk of a common phenotype or variants assessed within a clinical setting as possibly causing a monogenic disease (which would be most ClinVar variants).

The reviewer highlights an important distinction underlying monogenic disease and common trait variant effects. While our analysis has implications for both, we agree that the implications are different. We have expanded on this in the introduction and discussion sections (line 525). We have also made clearer distinctions between ClinVar and GWAS variants in the section the reviewer has highlighted (paragraph starting at line 257).

10. Linked to the point above, odds ratios for disease-associated variants would be useful to give an indication of the predicted impact of the variants on disease risk.

We have added a new heatmap to show the odds ratios for variants in the GWAS catalog using the median OR for protective and risk effects. We have shown this as a heatmap for simplicity providing the OR for all GWAS catalog variants, and within their trait categories (figure S3A). In most cases no significant difference in OR is observed between variants mapping to ref-exons or alt-exons.

In line with comments from reviewer 1, we additionally show new plots using fine-mapped variants from 14 traits (All of Us), and 94 traits (UK BioBank), to show the beta (against PIP), to illustrate the effect size of variants in ref-exons and alt-exons (figure S3C).

11. The authors may want to consider moving Panels E and F from figure 2 to the supplementary, the size of the panels make the variant position hard to interpret and the nature of the protein region that the variant is found in is clear from the text.

As suggested, these panels have been moved to a supplementary figure.

12. The ΔΔG values are somewhat difficult to interpret without a reference - it would be useful to have a range of values for known pathogenic missense variants in reference transcripts to compare it to.

Comparing variant effects in alterative isoforms, to those in reference isoforms can be challenging, as predictors can behave variably dependent on the underlying method. For example, ESM-1v utilises conservation, which does not permit a fair comparison. As the reviewer suggests, the AF3 method to derive ΔΔG values is more suitable. We provide additional analysis of ΔΔG values for pathogenic variants in reference isoforms, compared to VUS in alternative isoforms, for eight genes. In aggregate, variants mapping to alt-exons fall with the ΔΔG range of pathogenic variants in reference isoforms, albeit, and expectedly, with a lower median. We provide this as figure S4B, and discuss this in the results, on lines 250.

Reviewer #4 (Remarks to the Author):

This is a very beautiful and comprehensive piece of work that brings to light an important missing piece in the interpretation of disease-associated variants. I think the authors did an excellent job at presenting a very complex study, and I only have some very minor comments on the manuscript. I also have some comments on the figures that I believe would improve the final version of the paper.

Comments on manuscript
I think that there are quite a few methodological details that are brought into the Results section, and perhaps could be removed to help with readability.

The main text and figure legends have been refined to remove details present in the methods section.

Could the authors comment on how much this paper is shifting the paradigm that most GWAS variants are non-coding?

The reviewer raises an important point. While alt-exons would typically be classified as, for example, “intronic”, and therefore presumed non-coding in many annotations, by reclassifying the mapping to include alt-exons, this reclassifies 18.7% of GWAS catalog variants (EIC [11.9%] and ENIC [6.8%] alt-exons). Compared to ref-exons (7.5% of GWAS catalog) the reclassification of 18.7% from “non-coding” to alt-exons represents an important fraction, altering the interpretation of potential underlying mechanisms. Nevertheless, the majority (74%) of GWAS catalog variants, even after classification, remain non-coding. We have added these details and discussed the implications for non-coding variants in GWAS to the discussion (line 457).

line 120: are all the transcripts included here combined from public data?

All transcripts are from a publicly available dataset (FLIbase; Shi et al., 2024). This detail has been added to this sentence to clarify.

line 135: What is the distribution of alternative exons only? How many per gene?

In place of figure S1B, we have provided this as new figure S1A, showing the distribution of number of Ref- and Alt-exon classes - EIC and ENIC exons, per gene. The median number of exons per gene are similar across classes (Ref-exon = 8, EIC = 7, ENIC = 9).

line 140: too much detail for a results section?

These details have been removed, and the sentence simplified.

line 181: it would be interesting to compare this figure (~40K variants in alt-exons) to the number of variants found in ref-exons to give it more context.

Similar to the above comment about non-coding variants, we find ~24K map to ref-exons. We have added this to the results section (line 189).

lines 185-187: is this sentence referring to the right figure? Why was this performed for respiratory and endocrine traits specifically?

Thank you, the reference to the figure was incorrect, and refers to fig. 2A. This has been corrected. We chose to show respiratory and endocrine traits, with haematological traits in the supplemental, as they span a spectrum between enrichment scores of two to three-fold, while also representing traits likely to occur in tissues with RNA-seq available from GTEx.

lines 198-200: is this sentence referring to the right figure?

The figure layout has been changed throughout and this has been corrected.

line 531: do the AF bins include any of the values? For example, does the “common” bin include the 0.05 or 0.5 values?

We have corrected the descriptors to accurately reflect the inclusion of the boundary values where appropriate for the bin. This has also been corrected in the legend to figure 1C.

Figures
While I appreciate that there is a lot that needs to be summarised in this paper, I think there are too many panels for each figure, making them difficult to digest. Also there is a lot of back and forth between main and supplementary figures, so maybe the authors could reflect again on how to help the reader navigate this by perhaps moving some of them. I am making some more specific suggestions below.

We have made extensive changes to the panels and figures. There are now five main figures (previously four, with fewer panels per figure), and there are now 12 supplementary figures (previously 4), and seven supplementary tables (previously 2). We hope this improves the readability of the manuscript.

Fig. 1:
the text is very small, difficult to read even when zoomed in
panels B and D could be tables, leaving space for the rest of the figure

We have corrected this. Panel 1B has now been replaced as supplementary table S2. We have opted to keep panel 1D as a figure for visualisation purposes.

Fig. 2:
panel C can be a table

Figure 2C has been moved to a supplementary table S5.

Fig. 3
I would suggest moving panel C to supplementary materials

This has been moved to a supplementary figure.

Fig. 4

panel A can be a table
panel D is not cited in the main text

We have opted to keep figure 4A as a figure panel for visualization purposes of the tissue-specificity. The citation to 4D has been corrected.

Fig. S1
panels A and E could be tables

Panels S1A and S1E have been changed to supplementary tables S3 and S4, respectively.

Fig. S2
panel E in completely unreadable and panel A is not much better

These have been rearranged, with S2 now as new supplementary figures.

Fig. S3
panel D can be a table
panel E is difficult to read

Panel S3D has now been changed to include gnomAD variants mapping to the alternative isoform as a comparator, alongside VUS variants mapping to the alt-exon. As such, a table would no longer be feasible. The legibility of panel S3E has been improved. Text sizes have been increased, and in line with reviewer comments, we have included the reference isoform expression as discussed in response to reviewer #1.

Fig S4
panel A can be just described in the text
panel E would it be worth commenting on the correlation between different cell lines?

Panel S4A has now been removed and described in the text. A similar new analysis for fine-mapped UK biobank and All of Us variants, has also been described in text, rather than provided as a figure.
The correlation between cell lines shown in panel S4E (now figure S8C) is discussed in text, and likely arises due to the low number of target exons and genes in the panel.

Reviewer #1 (Remarks to the Author):

I thank the authors for their thorough and gracious response to my comments. The revised manuscript is substantially improved, and the authors have addressed all of my major and minor concerns & reservations about the analyses. I am satisfied with the revisions and recommend acceptance of the manuscript. Congratulations to the authors on a very interesting study that i'm sure will be of broad interest and utility to the genetics community!

One extremely tiny typographic correction: in the discussion the DPP9 variant is referred to as "chr19:1717660-A>G" rather than "chr19:4717660-A>G", which is used consistently throughout the rest of the manuscript. This should be corrected prior to publication.

Response: We thank the reviewer for their complements. We have corrected the error noted by the reviewer.

Reviewer #2 (Remarks to the Author):

We are happy with the changes that the authors made in response to our comments. We believe the manuscript is now more clear and do not have any further comments. We would like to congratulate the authors on their work.

Response: We thank the review for their input.

Reviewer #3 (Remarks to the Author):

I co-reviewed this manuscript with one of the reviewers who provided the listed reports. This is part of the Nature Communications initiative to facilitate training in peer review and to provide appropriate recognition for Early Career Researchers who co-review manuscripts.

Response: We thank the reviewer for their contribution.

Reviewer #4 (Remarks to the Author):

I think the paper has improved in readability thanks to all the changes implemented following the reviewers' comments. My concerns have been addressed and I am satisfied with the modifications and the answers provided.

Response: We thank the reviewer for their input.
